# Solvent-induced selectivity of Williamson etherification in the pursuit of amides resistant against oxidative degradation†

**DOI:** 10.1039/d0ra04465b

**Published:** 2020-06-25

**Authors:** James B. Derr, John A. Clark, Maryann Morales, Eli M. Espinoza, Sandra Vadhin, Valentine I. Vullev

**Affiliations:** Department of Biochemistry, University of California Riverside CA 92521 USA vullev@ucr.edu; Department of Bioengineering, University of California Riverside CA 92521 USA; Department of Chemistry, University of California Riverside CA 92521 USA; Materials Science and Engineering Program, University of California Riverside CA 92521 USA

## Abstract

This article reports two discoveries. (1) 2-Methoxyethanol induces unprecedented selectivity for etherification of 5-hydroxy-2-nitrobenzic acids without forming undesired esters. (2) Such compounds are precursors for amides showing unusual robustness against oxidative degradation, essential for molecular electrets that transfer strongly oxidizing holes at about −6.4 eV *vs.* vacuum.

## Abstract 2

Selective etherification produces precursors for amides resistant to oxidative degradation, *i.e.*, showing reversible oxidation at 1.5 to 1.7 V *vs.* SCE.

## Graphical abstract

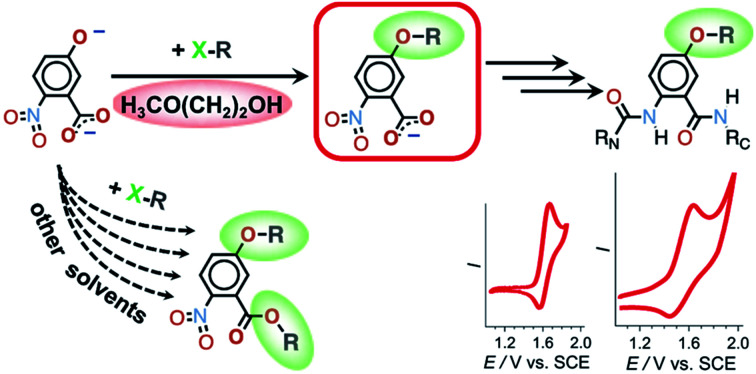

## Introduction

Williamson etherification (WE), involving alcohols and alkanes with good leaving groups as starting materials, remains the most broadly used method for the preparation of ethers.^1^ WE offers an important means for adding electron-donating groups to aromatic conjugates, essential for preparing electron-rich *p*-conducting organic materials. Despite its immense importance, the lack of selectivity presents challenges for the utility of WE.^1*c*^

**Chart 1:**
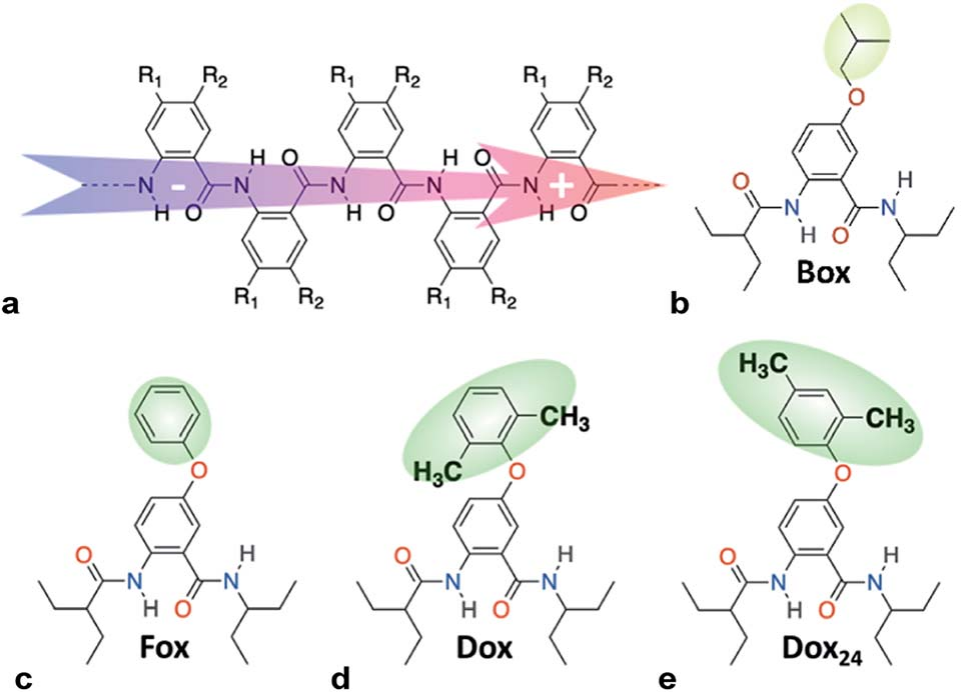
(a) Molecular anthranilamide (Aa) electret with its permanent electric dipole. (b–e) Electret Aa residues with either side chains that are highlighted.

To harness dipole effects on charge transfer (CT),^2^ we develop bioinspired molecular electrets that are based on polypeptide structures composed of anthranilamide (Aa) residues.^3^ (Electrets are systems with ordered electric dipole moments.) The amide and hydrogen bonds of the electrets are a source for their intrinsic dipole moments.^4^ The side chains of Aa, R_1_ and R_2_ (Chart 1a), on the other hand, provide a means for controlling their electronic properties.^4*a*,5^ Indeed, electric dipoles present key paradigms for controlling CT, which is of tremendous importance for energy conversion and organic electronics, among many other fields.^2*a*^ Molecular dipoles rectify CT,^2*b*,2*c*,3*c*,3*d*^ affecting charge separation and charge recombination.^3*c*^

Electron-donating side chains afford hole-transfer electrets.^5*d*^ For long-range CT in organic materials, it is important to attain a hopping (or incoherent) mechanism, for which the kinetics exhibits negligible distance dependence beyond about 1 nm.^6^ In order to prevent oxidative degradation of electrets mediating such hole hopping, it is crucial for the comprising Aa residues to form stable radical cations, Aa^•+^.^5*a*–*c*^ For attaining such stability, we have determined that: (1) the spin density distribution (SDD) of Aa^•+^ should not extend over its C-terminal amide;^5*b*^ and (2) the reduction potentials for oxidizing Aa, should not be too large, *i.e.*, *E*_Aa•+|Aa_ < 1.5 V *vs.* SCE, to prevent the inherent oxidative degradation of the amides.^5*b*^ The latter places a limit on how oxidizing the transferred holes can be. Hole hopping along moieties with as positive *E*_Aa•+|Aa_ as possible ensures the potency of the holes for attaining large open-circuit voltages and for driving chemical transformations. Because placing alkoxy side chains on Aa residues brings the reduction potentials of their radical cations, Aa^•+^, to the limit of 1.5 V *vs.* SCE,^5*a*–*c*^ such ether conjugates present a key paradigm in the pursuit of organic derivatives that can transduce strongly oxidizing charge carriers.

Established peptide-synthesis procedures, used on aromatic β-amino acids with protected amines, lead to non-reactive cyclic intermediates, preventing the formation of Aa derivatives.^5*f*,7^ Therefore, in the synthesis of Aa conjugates, we introduce each residue as its 2-nitrobenzoic acid (NBA) analogue.

**Scheme 1:**
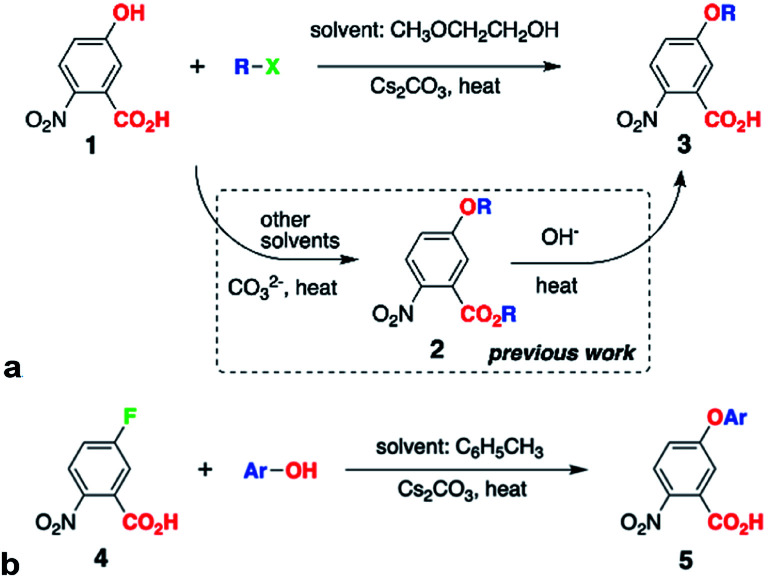
Etherification of 2-nitrobenzoic acid (NBA) derivatives. (a) –X = –Cl, –Br, –I; –R = –(CH_2_)_3_CH_3_, –CH_2_CH(CH_3_)_2_, –CH(CH_3_)C_2_H_5_, –C(CH_3_)_3_; for direct conversion of 1 to 3, 1 equiv. 1, 4 equiv. RX and 3 equiv. Cs_2_CO_3_; microwave heating: 2× 30 s, 60 W, temperature set to 130 °C, and ambient-pressure control; or conventional heating: 12 h, pressure tube at 130 °C; (b) –Ar = –C_6_H_5_, –2,6-C_6_H_3_(CH_3_)_2_ and −2,6-C_6_H_3_(CH_3_)_2_; 1 equiv. 4, 2–4 equiv. ArOH and 3–6 equiv. Cs_2_CO_3_; conventional heating: 12 h, pressure tube at 110 °C.

WE offers a means for synthesizing alkyloxy derivatives of NBA from alkyl halides and the corresponding NBA hydroxy analogues, 1 (Scheme 1a).^5*a*^ Because the carboxyl of 1 has a smaller p*K*_*a*_ value than the hydroxyl, both are deprotonated under the basic conditions of WE and can act as nucleophiles, leading to the formation of the esters of the alkoxy NBA derivatives, 2 (Scheme 1a).^5*a*^ This lack of selectivity of WE sets the requirement for an additional step, *i.e.*, hydrolysis, for obtaining the free-acid precursor, 3, for Aa synthesis (Scheme 1a).^5*a*^

Herein, we present the discovery of conditions for WE that allow for selective etherification of 1 without forming the undesired esters. Employing 2-methoxyethanol (2ME) as a solvent for WE of 1 with 1-iodobutane under microwave irradiation, affords 5-butoxy-2-nitrobenzoic acids, 3, in quantitative yields (R = *n*-C_4_H_9_, Scheme 1a, Table 1). Reacting 1 with other butyl halides under the same conditions, also results in direct production of 3 without formation of the ester, 2, or other side products. Reversing the WE paradigm, by taking advantage of the propensity of electron-deficient NBA fluorides to undergo nucleophilic aromatic substitution, provides routes to phenoxy NBA derivatives (Scheme 1b). As examined with cyclic voltammetry (CV), the electrochemical oxidation of the butoxy Aa derivatives barely shows any reversibility at scan rates of about 0.1 V s^−1^ or less. Conversely, some of the phenoxy Aa conjugates show not only improved chemical reversibility, but also *E*_Aa•+|Aa_ exceeding 1.5 V *vs.* SCE by about 200 mV. This result appears surprising because aliphatic and aromatic carboxamides readily undergo irreversible oxidative degradation at about 1.5 V *vs.* SCE.^8^ Therefore, these ether Aa conjugates have the potential to transduce strongly oxidizing holes making them comparable to a wide range of inorganic CT materials.

**Table 1:** Reaction yields of direct formation of 3 from 1 and X–R^a^

–R =	X =		
	I	Br	Cl
–(CH_2_)_3_CH_3_	97%	34%	15%
–CH_2_CH(CH_3_)_2_	40%	19%	Trace^b^
–CH(CH_3_)CH_2_CH_3_	41%	Trace^b^	Trace^b^
–C(CH_3_)_3_	Trace^b^	Trace^b^	N.D.^c^

a Yields are based on isolated and purified products, 3 (Scheme 1a).

b Products are not apparent on TLC but are detected using HRMS.

c Not detected with HRMS.

## Results and discussion

### Attaining selectivity for Williamson etherification

Others and we have shown the two-step synthesis of 3, starting with the preparation of 2 using polar solvent media at about 150 °C (Scheme 1a).^5*a*,9^ To lower the temperature and considerably shorten the reaction times, we resort to microwave radiation instead of conventional heating. Microwaving mixtures of 1 and various butyl halides dissolved in different solvents that are traditionally used for this reaction, such as *N,N*-dimethylacetamide and *N,N*-dimethylformamide (in the presence of Cs_2_CO_3_), does not produce even detectable amounts of either 2 or 3, as confirmed with high-resolution mass spectrometry (HRMS) of the reaction mixtures.

Polar solvents, however, are not usually beneficial for microwave-driven reactions. Medium polarity and hydrogen bonding can, indeed, stabilize transition states and lower activation energies. In such reaction mixtures, however, the polar solvents, rather than the reactants, tend to be the principal absorbers of the microwave energy. Conversely, non-polar solvents with high boiling points ensure that reactants absorb substantial amount of the microwave radiation. This direct transfer of energy to the reactants is a principal advantage that microwave-driven chemical conversions have over reactions under conventional heating.

Using low-polarity solvents, such as toluene and ethyl acetate, allows the formation of only traces of 3, as detected with HRMS. This minute conversion of 1 to 3 renders it impractical. Medium-polarity aprotic solvents, such as tetrahydrofuran (THF) and halogenated alkanes, do not produce even traces of 2 or 3. This lack of conversion extends even to the neat-reaction conditions, *i.e.*, when the butyl halide reactant is the solvent.

Conversely, when 2ME is the solvent for the same WE reaction of 1 with butyl halides, we observe the formation solely of the free acid, 3, as NMR analysis, *i.e.*, NOESY, confirms (see ESI†). Using other alcohols as solvents, such as isopropanol, also affords traces of 3 without 2, as HRMS reveals. While employing ethylene glycol for WE results in chromatographically detectable amounts of 3, the minute yields of a few percent renders it impractical. Ether solvents, such as THF and 1,2-dimethoxyethane, conversely, do not mediate the conversion of 1 to 3. While similar to ethylene glycol and 1,2-dimethoxyethane (R′O–CH_2_CH_2_–OR′′, R′ and R′′ = H or CH_3_), 2ME provides a unique reaction environment that under microwave radiation allows for etherification of the phenol OH of 1, without the side reaction of esterification of the carboxyl. This finding of the 2ME-mediated selectivity of WE is a key breakthrough of this study, eliminating the need for the second step, *i.e.*, hydrolysis of 2 (Scheme 1a).

As a variation of WE, Purdie–Irvine alkylation, involving Ag_2_O as a reagent with high affinity for the formed halide ions, allows for monomethylation of diols.^10^ Cs^+^ are the only available metal ions in the WE for converting 1 into 3 (Scheme 1a), and they do not have the chelation properties of Ag^+^. Conversely, strongly basic conditions involving treating alcohols with NaH or Na, and excess of these alkoxides when reacting them with chloro- or bromo-acetic acid, produce ethers capped with free carboxylates.^10*c*,11^ Such conditions keep the media dry and the dryness of the base is important for enhancing the WE yields.^12^ In the contrary to the previously reported synthesis of ethers with free carboxylates, we use: (1) a relatively mild base, Cs_2_CO_3_, (2) a hydroxyl-carboxyl starting material, 1, and (3) halides in four-fold excess (Scheme 1a). In addition to its selectivity, all these features represent key advantages of the 2ME-mediated WE.

The reaction yields of the direct synthesis of 3 from 1 manifest dependence not only on the halide, but also on the alkyl chain. Improving the leaving group, by varying the starting materials from chloride to bromide and iodide, increases the reaction yields (Table 1). Concurrently, branched primary butyls, X–CH_2_CH(CH_3_)_2_, show lower yields than the linear ones, X–(CH_2_)_3_CH_3_. The yields decrease further for secondary butyls, X–CH(CH_3_)C_2_H_5_; and for tertiary, X–C(CH_3_)_3_, we observe only traces of 3 or no product at all (Table 1). These effects of the halides and the alkyl chains on the yields are consistent with the S_N_^2^ mechanism expected for such WE reactions. In addition, the inherent instability of *t*-butyl ethers of electron-deficient phenols reflects the negligible and no yields of 3 when R = C(CH_3_)_3_.

The cleanliness of this WE reaction represents a principal advantage. Reacting 1 with 1-iodobutane produces the *n*-butyl analogue of 3 in quantitative yields (Table 1). For the other butyl halides the isolated-product yields are under 50%. Nevertheless, the reason for these yields was a lack of conversion despite the left over starting material 1, rather than a production of side products. Increasing the reaction times does not increase the ratio between 3 and 1, and does not improve the yields.

Transferring this microwave synthetic procedure to pressure-tube settings provides the means for scaling up and for using less expensive conventional heating sources. Heating for 12 hours at 130 °C, *i.e.*, about 5° above the boiling point of 2ME under normal pressure, results in similar selectivity of the production of 3. Monitoring the progress of the reaction, using TLC and HRMS, reveals formation of the ester 2, but with isolated yields that do not exceed 1%. Often, 2 disappears with extended reaction times. This finding suggests that 2ME has the perfect proticity resulting in pH of the reaction mixture that ensures the ether formation, but makes the ester unstable. Even if the ester 2 forms, the traces of water that build up with the progress of the reaction can drive its hydrolysis.

Switching the WE pattern allows for pursuing the synthesis of phenoxy Aa precursors. Instead of starting with halide derivatives of the side chains (Scheme 1a), we introduce the side chains as the WE nucleophiles (Scheme 1b). Fluorines on electron-deficient aromatic rings are immensely susceptible to nucleophilic aromatic substitution. Hence, introducing the side chain as the corresponding phenolate to fluorinated NBA, 4, allows for making the precursors, 5, for phenoxy Aa residues (Scheme 1b). The reaction yields for the phenoxy and 2,4-dimethylphenoxy analogues of 5, *i.e.*, the precursors for Fox and Dox_24_ (Chart 1c and e), are about 90%. The synthesis of the precursor of Dox, however, proceeds with yields smaller than 45%, most likely because of the steric hindrance induced by the two methyl groups at the ortho positions of the phenoxy side chain (Chart 1d).

Attempts to employ this nucleophilic aromatic substitution (Scheme 1b) for the synthesis of alkoxy Aa derivatives prove unfeasible. While the formation of the alkoxide ions, using metal sodium or strong imide bases, is quite trivial, 4 appears unstable under such extremely basic conditions.

### Oxidation reversibility at relatively high potentials

Converting the ether NBAs (3 and 5, Scheme 1) to their amide analogues capped with alkyls (Chart 1b and e), allows for examining the electrochemical properties of these Aa residues.^5*a*–*c*,5*e*^ We select one aliphatic and three aromatic ether derivatives: Box (with isobutyl), Fox (with phenyl), Dox (with 2,6-dimethylphenyl) and Dox_24_ (with 2,4-dimethylphenyl) (Chart 1b and e). In Dox and Dox_24_, the *ortho* and *para* positions of the electron-donating methyls ensure strong electronic coupling with the Aa ring. When both methyls are *ortho* to the oxygen, they can enforce orthogonality between the two aromatic rings and impede rotations around the ether linker, which represent the principal difference between Dox and Dox_24_ (Chart 1d and e).

**Fig. 1:**
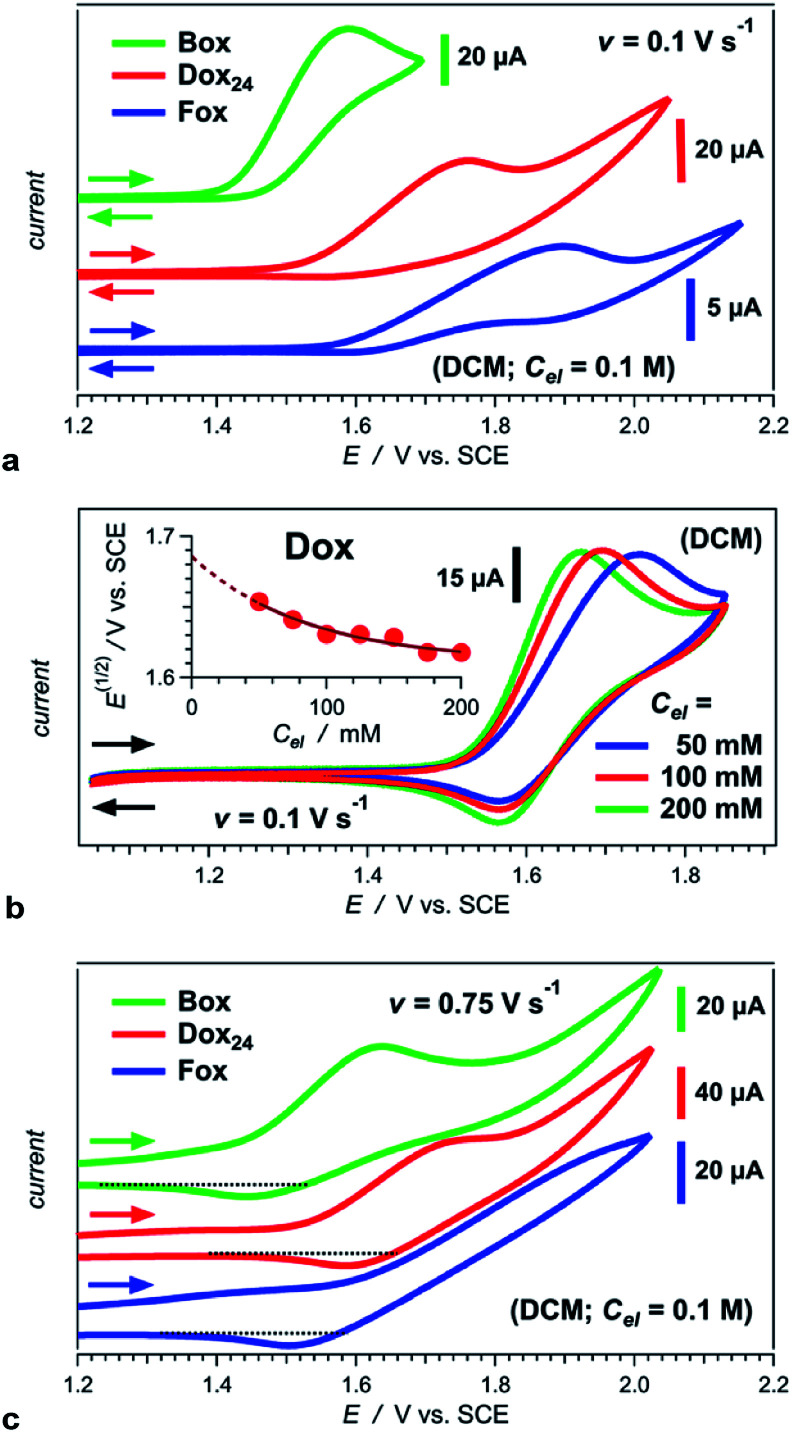
Cyclic voltammograms of Aa ether residues (Chart 1b and e) for DCM in the presence of an electrolyte, N(*n*-C_4_H_9_)_4_PF_6_, with varied concentration, *C*_el_. (a) Irreversible oxidation of Box, Fox and Dox_24_ at scan rate, *v* = 0.1 V s^−1^, and *C*_el_ = 100 mM. (b) Oxidation of Dox showing reversibility at 0.1 V s^−1^. Inset: dependence of the half-wave potential on *C*_el_ and extrapolation of its value for neat DCM, *i.e.*, for *C*_el_ = 0 M. (c) Partial reversibility of the oxidation of Box, Fox and Dox_24_ at 0.75 V s^−1^.

At moderate scan rates, Box exhibits irreversible oxidation at about 1.5 V *vs.* SCE for dichloromethane (DCM) media (Fig. 1a), which is consistent with the behaviour of other Aa residues with alkyloxy side chains.^5*a*^ Replacing the aliphatic with aromatic ether, methylated at the two *ortho* positions, leads to reversible oxidation at potential of about 1.7 V *vs.* SCE (*i.e.*, Box*vs.*Dox, Fig. 1b and Table 2), which is unprecedented for amides.

**Table 2:** Reduction potentials and optical excitation energies of Aa ether residues

	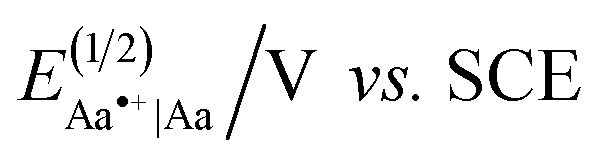 a		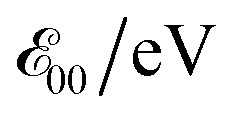 b	
	DCM	MeCN	DCM	MeCN
Box	1.56 ± 0.05	1.42 ± 0.07	3.48	3.52
Fox	1.82 ± 0.03	1.57 ± 0.02	3.53	3.56
Dox	1.69 ± 0.02	1.55 ± 0.03	3.52	3.55
Dox_24_	1.70 ± 0.04	1.56 ± 0.04	3.51	3.55

a Values for neat solvents, *i.e.*, extrapolated to *C*_el_ = 0 M (Fig. 1b) from cyclic voltammograms recorded at 0.05 and 0.1 V s^−1^. For irreversible oxidation, the values of the half-wave potentials at various *C*_el_ are obtained from the inflection points at the rise of the anodic peaks.^13^

b Estimated from the wavelengths, *λ*_00_, where the normalized absorption and fluorescence spectra cross, 
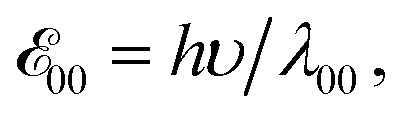
^5*c*,14^ which is a rough approximation for the transition-dipole-moment representation.^14*a*^

At the same scan rates of 0.1 V s^−1^ and smaller, the other two aromatic-ether residues, Fox and Dox_24_, exhibit irreversible oxidation similar to Box. Like Dox, however, Fox and Dox_24_ oxidize at more positive potentials than Box (Fig. 1a).

Increasing the scan rates beyond 0.5 V s^−1^ leads to partial oxidation reversibility of Box, Fox and Dox_24_, as evident from the small cathodic peaks that appear in their voltammograms (Fig. 1c). These findings indicate that the lifetimes of their radical cations are in the order of 1 s, which is sufficiently long for most CT applications. This oxidation reversibility (even if only partial), at potentials as high as 1.7 and 1.8 V *vs.* SCE, reveals an important effect that ethers have on aromatic amides.

Employing acetonitrile (MeCN) as a solvent yields the same trends as DCM. As expected, the reduction potentials for MeCN are not as positive as those for DCM (Table 2).

The Aa residues with aromatic ethers are harder to oxidize than the one with an alkyloxy group. Adding two methyls to the phenyloxy substituents causes a slight negative shift in the reduction potentials, *i.e.*, Fox*vs.*Dox and Dox_24_ (Table 2). While the latter agrees with the methyl electron-donating properties, the former appears counter-intuitive assuming that the phenyloxy groups extend the π-conjugation of the residues. Nevertheless, the zero-to-zero energies, 
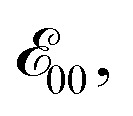
 of all ether residues are similar, around 3.5 eV (Table 2), regardless the aromatic *vs.* aliphatic nature of the substituent. It suggests that the resonance effect of the aromatic ethers on the π-conjugation does not necessarily dominate the electronic properties of the residues.

The Hammett constants and the field Swain–Lupton parameters of phenyloxy groups are more positive than those of alkyloxy substituents.^15^ When the conformations supress the resonance effects, allowing the inductive ones to dominate, the phenyloxy groups are stronger electron-withdrawers than the alkyloxy ones, which is consistent with our findings.

**Fig. 2:**
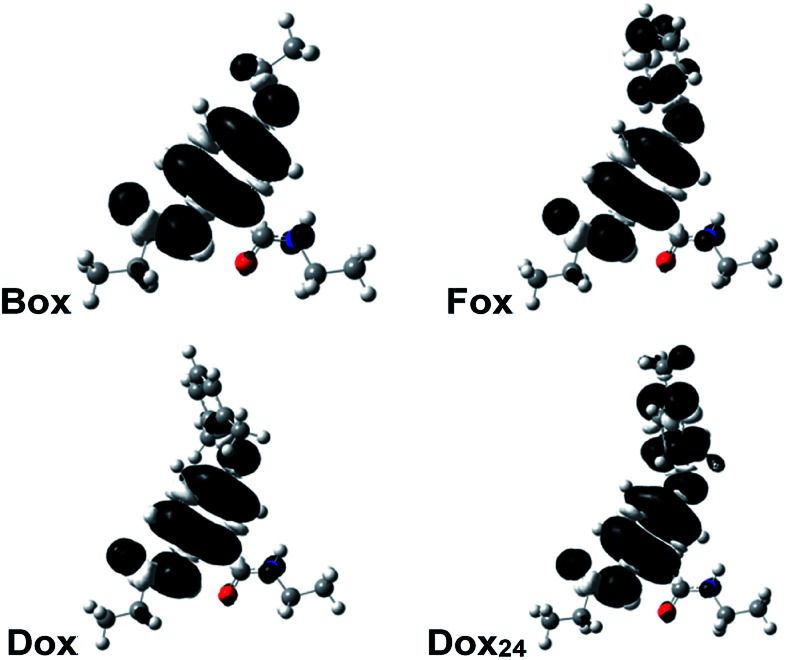
Spin-density distributions (SDDs) of the radical cations of the Aa ether residues (*anti* conformers) showing the delocalization of the positive charge. For the DFT computations, the alkyls chains are truncated to ethyls.

Why do the methyls at the *ortho* positions of the phenyloxy substituent provide extra stability of the radical cations of Dox? The sterics of the *ortho*-methyls forces orthogonality between the two aromatic rings of Dox, and SDD does not extend over the phenyl side chain. That is, Dox and Box manifest similar SDD that extends over the N-terminal amide and the ether oxygen (Fig. 2). Conversely, the aromatic rings of Fox and Dox_24_ are not orthogonal to each other, and the SDD expands over the phenyls of the side chains (Fig. 2).

These contrasts between SDD of Dox^•+^ and of the radical cations of the other two aromatic ethers could account for the difference between the reversibility of their oxidation. Conversely, Dox shows reversibility at low scan rates, while Box does not. Yet, they have quite similar SDDs (Fig. 2).

Two conformers of Dox provide orthogonality between its aromatic rings: *i.e.*, with the phenyloxy at *syn vs. anti* orientation in relevance to the C-terminal amide. While the computed energy differences between the *syn*- and *anti*-Dox are comparable to the thermal energy, *k*_B_*T* at *T* ≈ 20 °C, the NMR spectra show a single set of peaks for the 2,6-dimethylphenyloxy protons. We ascribe it to an interexchange between the two conformers that is faster than the NMR acquisition timescales. The structural features of Dox make the electronic properties of its radical cation similar to those of Box^•+^, rather than Fox^•+^ and Dox_24_^•+^. Unlike Box^•+^, however, Dox^•+^ has a polarizable aromatic side chain.

This strategy for decreasing the susceptibility of aromatic amides to oxidative degradation by adding ether substituents is considerably more attractive than an alternative means involving modification of their –CONH– groups. Introducing capping groups with quaternary carbons that connect to the amides of Aa residues does not detectably improve the stability of their radical cations. Oligomers of Aa residues that oxidize irreversibly, still show irreversible voltammograms.^5*f*^ Conversely, converting the C-terminal amide, Aa–CONH–R, to a tertiary amide, *i.e.*, to Aa–CONR_2_, induces partial reversibility in the voltammograms of residues that oxidize irreversibly.^5*b*^ It is consistent with the importance of the proton of the C-terminal amide for the pathways of oxidative degradation. In an Aa oligomer (Chart 1a), however, the introduction of such tertiary amides along its back bone would disrupt the hydrogen-bonding pattern that is important for its extended conformation and macrodipole. The C-terminal protons of such oligomers are not involved in the hydrogen bonding network, as NMR studies show.^3*b*^ Therefore, capping the C-terminus of an Aa oligomer with tertiary amide will not disrupt its conformational integrity. Still, computational analysis show that the positive charge of a singly oxidized oligomer, composed of identical Aa units, localizes on the N-terminal residue. Therefore, tertiary amide on C-terminus would prove beneficial only if the hole transfer away from the C-terminal residue is slower than its oxidative degradation. In light of this discussion, gaining oxidation reversibility *via* adding ether side chains is a superb strategy because it does not involve disruption of the amides along the backbone that maintain the extended secondary conformation of the Aa oligomers.

## Conclusions

Ether substituents show important propensity for stabilizing oxidized amides. Ethers are strong enough electron-donating groups to remove the positive charge in the radical cations from the amide groups susceptible to oxidative degradation, but not strong enough to cause drastic negative shifts in reduction potentials. Orthogonality of the aromatic rings of such ether substituents is especially promising for the design of hole-transfer amides. Selectivity for etherification of aromatic hydroxyls in the presence of other nucleophilic groups is important for the synthesis of such charge-transfer conjugates that, along with their newly discovered electronic properties, opens new doors for organic electronics and energy science.

## Conflicts of interest

There are no conflicts to declare.

## Supplementary Material

RA-010-D0RA04465B-s001
