# Supplementary material for: Solvent-induced selectivity of Williamson etherification in the pursuit of amides resistant against oxidative degradation
Source: RSC Adv. 2020 Jun 25;10(41):24419–24. doi: 10.1039/d0ra04465b (PMC9055110; doi:10.1039/d0ra04465b)
Supplement: RA-010-D0RA04465B-s001 [file RA-010-D0RA04465B-s001.pdf]

Electronic Supplementary Material (ESI) for RSC Advances.
This journal is © The Royal Society of Chemistry 2020

Electronic Supplementary Information

Solvent-induced selectivity of Williamson etherification in the pursuit of amides resistant against oxidative degradation

James B. Derr,[a] John A. Clark,[b] Maryann Morales,[c] Eli M. Espinoza,[c,†] Sandra Vadhin,[b,‡] and Valentine I. Vullev*[a,b,c,d]

[a.] *Department of Biochemistry, University of California, Riverside, CA 92521, USA.*
[b.] *Department of Bioengineering, University of California, Riverside, CA 92521, USA.*
[c.] *Department of Chemistry, University of California, Riverside, CA 92521, USA.*
[d.] *Materials Science and Engineering Program, University of California, Riverside, CA 92521, USA.*
† Present Address: College of Bioengineering, University of California, Berkeley, CA 94720, USA.
‡ Present address: School of Chemical and Biomolecular Engineering, Cornell University, Ithaca, NY 14853, USA.

Table of contents

MATERIALS **S2**
- **General methods** **S2**
- **Synthetic procedures** **S2**
- **^{1}H and ^{13}C NMR spectra** **S14**
- **Proofs for the selective formation of ethers, rather than esters** **S28**
- ***Syn* and *anti* conformers of Dox** **S31**

METHODS **S32**
- **Cyclic voltammetry** **S32**
- **Steady-state optical spectroscopy** **S33**
- **Computational methods** **S34**

MATERIALS

General methods. All chemicals were used as received unless otherwise specified. The reported ^{1}H NMR, ^{13}C NMR, and NOESY spectra were recorded on 400 MHz, 500 MHz and 600 MHz spectrometers. ^{1}H chemical shifts (δ) are reported in ppm relative to $CHCl_3$ in $CDCl_3$ (δ = 7.26 ppm); ^{13}C δ are reported in ppm relative to $CDCl_3$ (δ = 77.23 ppm). Data for ^{1}H NMR are reported as follows: chemical shift, integration, multiplicity (s = singlet, d = doublet, t = triplet, q = quartet, p = pentet/quintet, h = hextet/sextet, e = eptet(from επτά)/heptet, m = multiplet), and coupling constants. All ^{13}C NMR spectra were recorded with complete proton decoupling. High-resolution mass spectrometry (HRMS) was performed using Agilent LCTOF (6200) mass spectrometer (Agilent Technologies, Santa Clara, CA). Analytical thin layer chromatography was performed using 0.25 mm silica gel 60-F plates. Flash chromatography was performed using 60 Å, 32−63 μm silica gel.

Synthetic procedures

Scheme S1. Synthesis of ether derivatives, **3** (Scheme 1a).

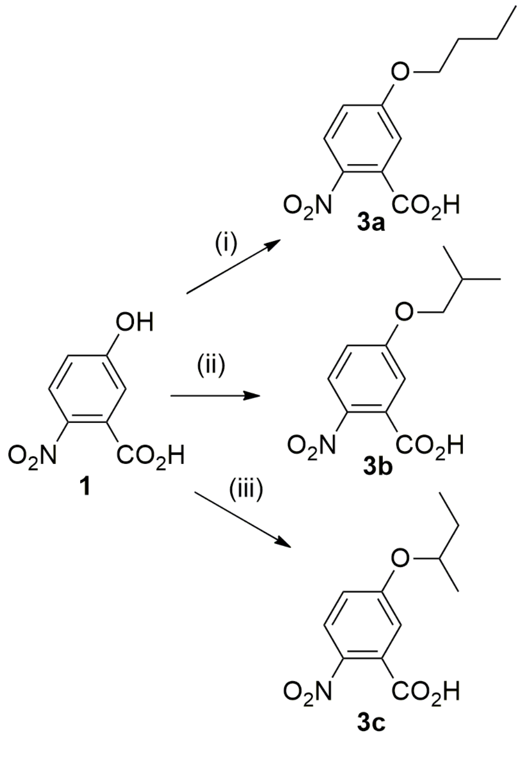

(i) C_4H_9X, Cs_2CO_3, 2ME, 130°C, 30 sec, 97%; (ii) C_4H_9X, Cs_2CO_3, 2ME, 130°C, 30 sec, 40%; (iii) C_4H_9X, Cs_2CO_3, 2ME, 130°C, 30 sec, 41%.

Scheme S2. Synthesis of Box.

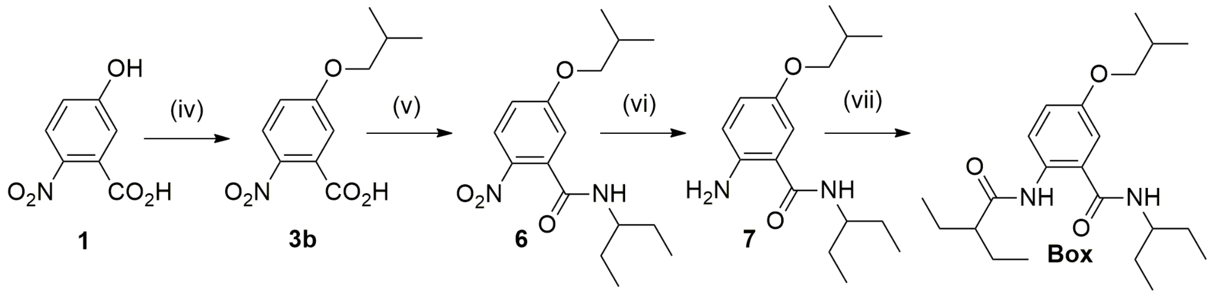

(iv) 1-iodo-2-methylpropane, Cs_2CO_3, 2ME, 130°C, overnight, 40%; (v) 3-aminopentane, HATU, HOAt, DIPEA, DMF, r.t., Overnight, 81%; (vi) Pd/C, H_2, Ethyl Acetate, r.t., Overnight; (vii) 2-ethylbutyric acid, HATU, HOAt, DIPEA, DMF, r.t., Overnight, 71%.

Scheme S3. Synthesis of Fox.

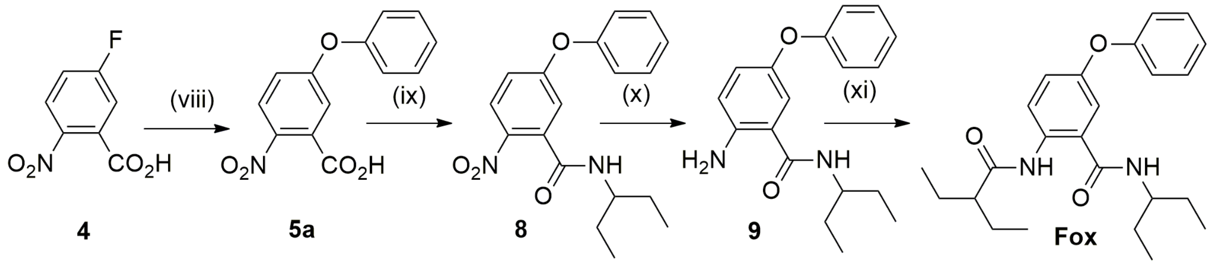

(viii) Phenol, Cs_2CO_3, Toluene, 110°C, Overnight, 92%; (ix) (1) $(COCl)_2$, DCM, DMF, -78 °C to r.t., 1 hr, 72%; (2) 3-aminopentane, DCM, NMM, -78 °C to r.t., 6h, 72%; (x) Pd/C, H_2, Ethyl Acetate, r.t., Overnight; (xi) 2-ethylbutanoyl chloride, DCM, NMM, -78 °C to r.t., 4h, 25%.

Scheme S4. Synthesis of Dox.

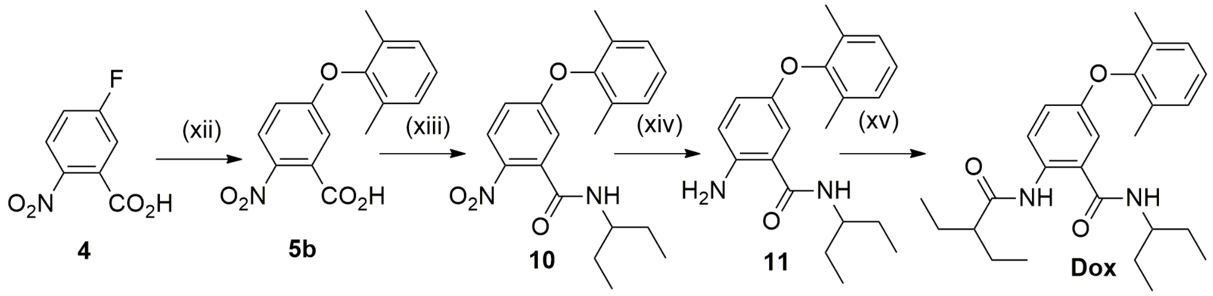

(xii) 2,6-Dimethylphenol, Cs_2CO_3, Toluene, 110°C, Overnight, 42%; (xiii) (1) $(COCl)_2$, DCM, DMF, -78 °C to r.t., 1 hr; (2) 3-aminopentane, DCM, NMM, -78 °C to r.t., 6h, 26%; (xiv) Pd/C, H_2, Ethyl Acetate, r.t., Overnight; (xv) 2-ethylbutanoyl chloride, DCM, NMM, -78 °C to r.t., 4h, 29%.

Scheme S5. Synthesis of Dox_{24}.

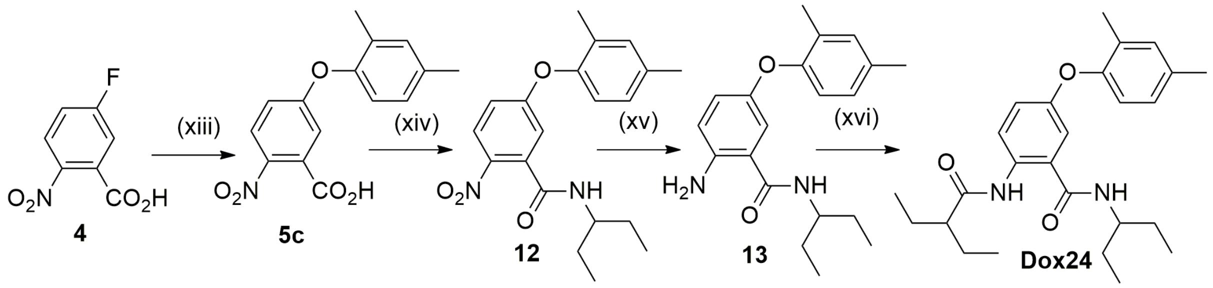

(xiii) 2,4-Dimethylphenol, Cs_2CO_3, Toluene, 110°C, Overnight, 88%; (xiv) (1) $(COCl)_2$, DCM, DMF, -78 °C to r.t., 1 hr; (2) 3-aminopentane, DCM, NMM, -78 °C to r.t., 6h, 64%; (xv) Pd/C, H_2, Ethyl Acetate, r.t., Overnight; (xvi) 2-ethylbutyric acid, HATU, HOAt, DIPEA, DMF, r.t., Overnight, 58%.

Optimization of solvent and reaction times. Our previous work on synthesis of alkyloxy NBA derivatives[5a] provides the initial choices of a base (i.e., Cs_2CO_3) and reactant equivalents (i.e., 4:1 equivalents of halide to **1**, except for neat reaction where we use the halide as a solvent). The microwave synthesis is conducted using a Discover LabMate reactor equipped with IntelliVent Pressure control (CEM Corporation, Matthews, NC, USA). The temperature limit is set for 130 ºC, and the power – to 60 W. The pressure control (set for ambient pressure) prevents the reaction mixture from overheating and the solvent from boiling out of the reaction vessel. We employ consecutive treatment of 30 seconds each. After each treatment, we examine the reaction mixture using TLC. After completing the reaction, use HRMS to examine the crude reaction mixture and each spot on the TLC (which we scrape off the TLC and extract with methanol for analysis with ESI HRMS).

As an example, for the synthesis of **3b**, from **1** and 1-iodo-2-methylpropane (Scheme S1), we examine a wide variety of solvents (Table S1). For a substantial number of the examined solvents, the TLC results

are identical for before and after the reaction (2×30 s microwave treatment), and the HRMS does not detect product, **3b**, in the crude reaction mixture. Reactions employing other set of solvents, mostly alcohols, yield traces of **3b**, detectable with HRMS from the crude mixture, and may or may not show additional spot on the TLC, identified with the product (Table S1). While reactions in ethylene glycol produce both, a small product spot on the TLC confirmed with HRMS, the overall isolated yields were less than 5%, which renders this solvent as unfeasible for further pursuits.

By far, 2-methoxyethanol (2ME) produces the highest yields (Table S1). As evident from the intensities of the product and starting-material spots on the TLC, even 30-s microwave treatments result in higher conversions for 2ME than for the other solvents for 2×30 s reaction times. An increase in the reaction times for 2ME to 60 s improves the conversion (Table S2). Additional increases in the reaction times, however, does not result in further improvements of the conversion, as the TLC analysis and the ratio between the intensities for the peaks for 3b and 1 show on the mass spectra show. The reactions of **1** with the other butyl halides reveal similar trends.

Table S1. Solvent optimization for direct conversion of 1 to 3b (Scheme 1a and S1).[a]

Solvent	Conversion
2-Methoxyethanol	Notable [b]
Methanol	Minimal [c]
Ethanol	Minimal
Isopropyl Alcohol	Minimal
Ethylene Glycol	Minimal
Dichloromethane	None [d]
1,2-Dichloroethane	None
1-Iodo-2-Methylpropane	None
1,2-Dimethoxyethane	None
N,N-Dimethylformamide	None
N,N-Dimethylacetamide	None
Tetrahydrofuran	None
Toluene	Minimal
Ethyl Acetate	Minimal

[a] Reaction conditions: substrate **1** (0.5 mmol), halide **2** (4 equiv.), Cs_2CO_3 (3 equiv.), dry solvent (1mL); two 30-second intervals of microwave treatment: temperature set to 130 °C at 60 W power, pressure sensor set for ambient pressure, under argon atmosphere. [b] Notable conversion: isolated product yields exceed 5%. [c] Minimal conversion: detectable with HRMS, but yields smaller than 5%; also may or may not be detectable on TLC. [d] No conversion: product not detected with HRMS.

Table S2. Optimization of reaction times for conversion of 1 to 3b (Scheme 1a and S1).[a]

time / s [b]	Conversion
30	Partial [c]
60	Complete [d]
90	No Further Conversion [e]
120	No Further Conversion
170	No Further Conversion

[a] Reaction conditions: substrate **1** (0.5 mmol), halide **2** (4 equiv.), Cs_2CO_3 (3 equiv.), dry 2ME (1 mL); Microwave treatment: temperature set to 130 °C at 60 W power, pressure sensor set for ambient pressure, under argon atmosphere. [b] The reaction time varies in increment of 30 s for the first 2 min, and additional 50 s after that. [c] Partial conversion: further microwave treatments lead to a visible increase in the ratio between **3b** and **1** (as determined by TLC and HRMS). [d] Complete conversion: further microwave treatments do not improve the ratio between **3b** and **1**. [e] No further conversion: no visible improvement in the ration between **3b** and **1**.

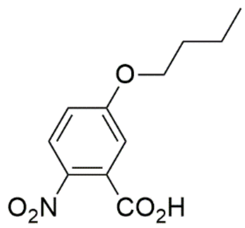

5-butoxy-2-nitrobenzoic acid (3a) (Scheme S1). Cs_2CO_3 (488 mg, 1.5 mmol) was placed in a microwave vial, and purged with argon. 5-hydroxy-2-nitrobenzoic acid, **1** (91.6 mg, 0.5 mmol), was added, followed by the 1-iodobutane (230 µL, 2.0 mmol) and 1mL of 2-methoxyethanol (2ME). The microwave vial was capped and put into the microwave. The parameters were set to 130°C, 60 W, 2×30 seconds. After the first interval of 30 s, the reaction mixture was allowed to cool for 5 minutes and then microwaved again at the same exact parameters giving a dark orange solution. The progress of the reaction was monitored via TLC. The mixture was diluted with 5% HCL and extracted with DCM (3×25mL). The organic layer was dried Na_2SO_4 and condensed. The product was purified using flash chromatography (stationary phase: silica gel; eluent gradient: 0:1 (v:v) to 1:1 (v:v) of ethyl acetate and hexanes. The 1:1 elution was mixed with 1% acetic acid. The product was condensed to afford 116 mg (97%) of **3a** (white crystals). ^{1}H NMR (600 MHz, Chloroform-*d*) δ 8.00 (d, *J* = 9.1 Hz, 1H), 7.17 (d, *J* = 2.7 Hz, 1H), 7.04 (dd, *J* = 9.1, 2.7 Hz, 1H), 4.08 (t, *J* = 6.5 Hz, 2H), 1.81 (dq, *J* = 8.7, 6.6 Hz, 2H), 1.55 – 1.45 (m, 2H), 0.98 (t, *J* = 7.4 Hz, 3H). ^{13}C NMR (151 MHz, $CDCl_3$) δ 171.56, 163.15, 140.13, 130.09, 126.88, 116.91, 114.96, 77.44, 77.23, 77.02, 69.22, 31.04, 19.25, 13.90. HRMS (ESI) m/z calculated for $C_{11}H_{12}NO_5$: $[M-H]^-$ 238.0721, found 238.0741.

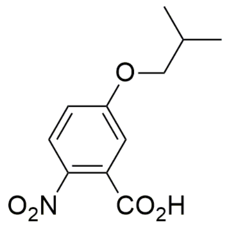

5-isobutoxy-2-nitrobenzoic acid (3b) (Scheme S1). *Microwave procedure:* Cs_2CO_3 (488 mg, 1.5 mmol) was placed in a microwave vial, and purged with argon. 5-hydroxy-2-nitrobenzoic acid, **1** (91.6 mg, 0.5 mmol), was added, followed by the 1-iodo-2-methylpropane (230 µL, 2.0 mmol) and 1 mL of 2ME. The microwave vial was capped and put into the microwave. The parameters were set to 130°C, 60 W, 2×30 seconds. After the first interval of 30 s, the reaction mixture was allowed to cool for 5 minutes and then microwaved again at the same exact parameters giving a dark orange solution. The progress of the reaction was monitored via TLC. The mixture was diluted with 5% HCL and extracted with DCM (3×25mL). The organic layer was dried Na_2SO_4 and condensed. The product was purified using flash chromatography (stationary phase: silica gel; eluent gradient: 0:1 (v:v) to 1:1 (v:v) of ethyl acetate and hexanes. The 1:1 elution was mixed with 1% acetic acid. The product was condensed to afford 116 mg (40%) of **3b** (tan powder). *Pressure-tube conventional-heating procedure*: **1** (3.7 g, 20 mmol) was placed in in a pressure tube with a stir bar, and purged with argon. Fifteen mL of 2ME was added, followed by the cesium carbonate (9.77 g, 30 mmol), and 1-bromo-2-methylpropane (4.3 mL, 40 mmol). The mixture was stirred at 130 °C overnight. The progress of the reaction was monitored via TLC. The reaction was taken out of the oil bath, allowed to cool, diluted with 200 mL of 5% HCl and extracted with DCM (3×25mL). Dried over Na_2SO_4 and vacuum filtered. The filtrate was condensed. The product was purified using flash chromatography (stationary phase: silica gel; eluent gradient: 0:1 (v:v) to 1:1 (v:v) of ethyl acetate and hexanes. The 1:1 elution was mixed with 1% acetic acid. The product was condensed to afford 901 mg (18%) of **3b** (tan powder). ^{1}H NMR (600 MHz, Chloroform-*d*) δ 8.02 (d, *J* = 9.1 Hz, 1H), 7.17 (d, *J* = 2.7 Hz, 1H), 7.05 (dd, *J* = 9.1, 2.7 Hz, 1H), 3.84 (d, *J* = 6.5 Hz, 2H), 2.13 (dq, *J* = 13.3, 6.7 Hz, 1H), 1.05 (d, *J* = 6.7 Hz, 6H). ^{13}C NMR (151 MHz, $CDCl_3$) δ 163.27, 140.13, 130.28, 126.92, 116.87, 114.97, 77.44, 77.23, 77.02, 75.71, 29.92, 28.34, 19.27, 19.14. HRMS (ESI) m/z calculated for $C_{11}H_{12}NO_5$: $[M-H]^-$ 238.0721, found 238.0827.

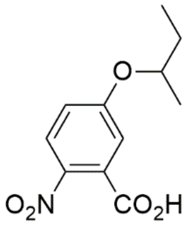

5-(2-*n*-butoxy)-2-nitrobenzoic acid (3c) (Scheme S1). Cs_2CO_3 (488 mg, 1.5 mmol) was placed in a microwave vial, and purged with argon. 5-hydroxy-2-nitrobenzoic acid, **1** (91.6 mg, 0.5 mmol), was added, followed by the 2-iodobutane (230 µL, 2.0 mmol) and 1mL of 2ME. The microwave vial was capped and put into the microwave. The parameters were set to 130°C, 60 W, 2×30 seconds. After the first interval of 30 s, the reaction mixture was allowed to cool for 5 minutes and then microwaved again at the same exact parameters giving a dark orange solution. The progress of the reaction was monitored via TLC. The mixture was diluted with 5% HCL and extracted with DCM (3×25mL). The organic layer was dried Na_2SO_4 and condensed. The product was purified using flash chromatography (stationary phase: silica gel; eluent gradient: 0:10 (v:v) to 1:1 (v:v) of ethyl acetate and hexanes. The 1:1 elution was mixed with 1% acetic acid. The product was condensed to afford 116 mg (41%) of **3c** (white crystals). ^1H NMR (600 MHz, $CDCl_3$) δ 10.33 (s, 1H), 8.01 (d, J = 9.1 Hz, 1H), 7.15 (d, J = 2.7 Hz, 1H), 7.02 (dd, J = 9.1, 2.7 Hz, 1H), 4.46 (h, J = 6.1 Hz, 1H), 1.79 (ddd, J = 13.8, 7.5, 6.3 Hz, 1H), 1.74 – 1.64 (m, 1H), 1.35 (d, J = 6.1 Hz, 3H), 0.99 (t, J = 7.5 Hz, 3H. ^{13}C NMR (151 MHz, $CDCl_3$) δ 171.72, 162.50, 139.80, 130.18, 127.01, 117.63, 115.77, 77.44, 77.23, 77.02, 76.83, 29.90, 29.13, 19.11, 9.78. HRMS (ESI) m/z calculated for $C_{11}H_{12}NO_5$: $[M-H]^-$ 238.0721, found 238.1227

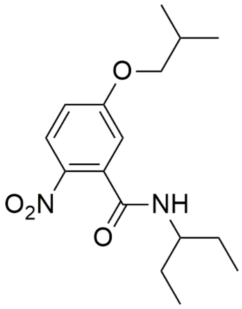

5-isobutoxy-2-nitro-N-(pentan-3-yl)benzamide (6) (Scheme S2). 3b (1.26 g, 5.29 mmol), 1-[Bis(dimethylamino)methylene]-1H-1,2,3-triazolo[4,5-b]pyridinium 3-oxide hexafluorophosphate (HATU) (2.8 g, 7.41 mmol), 1-hydroxy-7-azabenzotriazole (HOAt) (1.0 g, 7.41 mmol), and N-diisopropylethylamine (DIPA) (1.3 mL, 7.41 mmol) were dissolved in 15 mL of DMF in a dry, argon purged 100 mL round bottom flask with a stir bar. This was stirred for 15 minutes and then 3-aminopentane (860 µL, 7.41 mmol) was transferred and stirred overnight. The progress of the reaction was monitored via TLC. The mixture quenched with 100 mL of saturated Na_2CO_3 was extracted with DCM (3×50mL). The organic layer was dried Na_2SO_4 and condensed. The product was purified using flash chromatography (stationary phase: silica gel; eluent gradient: 0:10 (v:v) to 3:2 (v:v) of ethyl acetate and hexanes in 200 mL increments.to afford 1.39 g (81%) of **6** (white powder). ^1H NMR (500 MHz, Chloroform-*d*) δ 8.07 (d, J = 9.1 Hz, 1H), 6.92 (dd, J = 9.1, 2.7 Hz, 1H), 6.87 (d, J = 2.7 Hz, 1H), 5.56 (d, J = 9.1 Hz, 1H), 3.96 (dtt, J = 8.9, 7.5, 5.5 Hz, 1H), 3.80 (d, J = 6.5 Hz, 2H), 2.11 (hept, J = 6.7 Hz, 1H), 1.64 (dddd, J = 14.9, 13.0, 7.5, 5.5 Hz, 2H), 1.51 (dt, J = 13.9, 7.4 Hz, 2H), 1.06 – 0.96 (m, 12H). ^{13}C NMR (126 MHz, cdcl$_3$) δ 166.64, 163.65, 138.68, 136.33, 127.38, 114.88, 114.79, 77.48, 77.23, 76.98, 75.53, 53.05, 28.32, 27.24, 19.25, 10.40. HRMS (ESI) m/z calculated for $C_{16}H_{24}N_2O_4$: $[M+H]^+$ 309.1814, found 309.2099

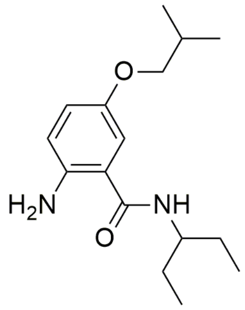

2-amino-5-isobutoxy-N-(pentan-3-yl)benzamide (7) (Scheme S2). 6 (1.28 g, 3.94 mmol), Pd/C (192 mg, 15% by weight), and 15 mL of ethyl acetate was transferred in a dry, Ar purged 100 mL round bottom flask with a stir bar. The round bottom was then vacuumed for 5 min. H_2 gas was attached to the round bottom and the reaction was stirred overnight. The reaction was then vacuumed filtered and condensed giving crude **7** (black oil). HRMS (ESI) m/z calculated for $C_{16}H_{25}N_2NaO_2$: $[M+Na]^+$ 300.1819, found 299.8578.

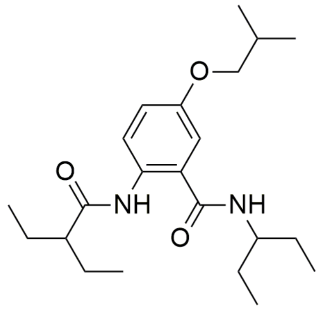

2-(2-ethylbutanamido)-5-isobutoxy-N-(pentan-3-yl)benzamide (Box) (Scheme S2). HATU (2.1 g, 5.52 mmol), HOAt (751 mg, 5.52 mmol), 2-ethylbutyric acid (696 µL, 5.52 mmol), and DIPEA (960 µL, 5.52 mmol) were dissolved in 15 mL of DMF in a dry, argon purged 100 mL round bottom flask with a stir bar. This mixture was stirred for 15 minutes and **7** (produced from 3.94 mmol of **6**) was transferred to it and stirred overnight. The progress of the reaction was monitored via TLC. The mixture quenched with 100 mL saturated Na_2CO_3 was extracted with DCM (3×50mL). The organic layer was dried Na_2SO_4 and condensed. The product was purified using flash chromatography (stationary phase: silica gel; eluent gradient: 0:10 (v:v) to 2:3 (v:v) of ethyl acetate and hexanes in 200 mL increments.to afford 1.06 g (71%) of **Box** (white powder). ^{1}H NMR (500 MHz, Chloroform-*d*) δ 10.49 (s, 1H), 8.46 (d, *J* = 9.1 Hz, 1H), 6.99 (dd, *J* = 9.1, 2.9 Hz, 1H), 6.94 (d, *J* = 2.9 Hz, 1H), 5.80 (d, *J* = 9.2 Hz, 1H), 3.96 (dtd, *J* = 13.6, 8.4, 5.2 Hz, 1H), 3.72 (d, *J* = 6.6 Hz, 2H), 2.13 – 2.01 (m, 2H), 1.68 (qdd, *J* = 12.7, 8.3, 6.2 Hz, 5H), 1.61 – 1.40 (m, 4H), 1.03 (d, *J* = 6.7 Hz, 6H), 0.94 (dt, *J* = 12.1, 7.4 Hz, 12H). ^{13}C NMR (126 MHz, cdcl$_3$) δ 174.74, 168.81, 154.70, 132.28, 123.59, 116.91, 113.38, 77.49, 77.23, 76.98, 75.21, 52.88, 52.85, 28.55, 27.79, 26.01, 19.45, 12.23, 10.54. HRMS (ESI) m/z calculated for $C_{22}H_{36}N_2O_3$: $[M+H]^+$ 377.5387, found 377.2986.

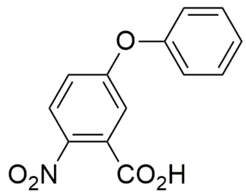

2-nitro-5-phenoxybenzoic acid (5a) (Scheme S3). 4 (3.5 g, 19 mmol) was placed in in a pressure tube with a stir bar, and purged with argon. 20 mL of dry toluene was added, followed by the cesium carbonate (19 g, 57 mmol), and phenol (7.1 g, 76 mmol). The mixture was stirred at 110°C overnight. The progress of the reaction was monitored via TLC. The reaction was taken out of the oil bath for 10 minutes to cool and then diluted with 200 mL of 5% HCl and extracted with DCM (3×25mL). Dried over Na_2SO_4 and vacuum filtered. The filtrate was condensed. The product was crashed out in DCM and Hexanes. The product was condensed to afford 4.52 g of (92%) of **5a** (white crystals). ^{1}H NMR (500 MHz, Chloroform-*d*) δ 8.03 – 7.97 (m, 2H), 7.52 (dd, *J* = 7.8, 2.8 Hz, 2H), 7.49 – 7.42 (m, 1H),

7.36 (ddd, J = 9.0, 7.2, 2.8 Hz, 2H), 7.15 – 7.08 (m, 1H). ^{13}C NMR (151 MHz, $CDCl_3$) δ 168.97, 162.26, 130.76, 129.87, 126.92, 126.09, 120.79, 119.39, 117.64, 115.21, 77.44, 77.23, 77.02.
HRMS (ESI) m/z calculated for $C_{13}H_8NO_5$: [M-H]$^-$ 258.0408, found 258.0438

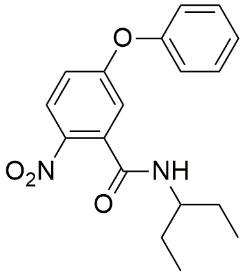

2-nitro-N-(pentan-3-yl)-5-phenoxybenzamide (8) (Scheme S3). 5a (1.1 g, 4.1 mmol) was transferred to a dry, Ar purged 100 mL round bottom flask, with a stir bar. This was dissolved in 20 mL of DCM, with 3 drops of DMF. The reaction mixture was cooled to -78 °C and was stirred for 5 minutes. Then, (1.4 mL, 16 mmol) of oxalyl chloride was added dropwise, the stirred for 1 hr The reaction was monitored via TLC by taking a few drops of reaction mixture and adding it to methanol. This would generate the ester from and move up the TLC plate. The reaction mixture was condensed 3 times, each time adding 20 mL of DCM. The condensed product was dissolved in 20 mL of DCM and cooled to -78°C for 5 min. 3-aminopentane (0.93 mL, 8 mmol) was added dropwise followed by *N*-methylmorpholine (NMM) (2.2 mL, 20 mmol). This was raised to room temperature and reacted overnight. Upon completion of the reaction, the mixture was quenched with 100 mL of 5% HCl. The mixture was extracted with DCM (3×25 mL). The organic layer was dried Na_2SO_4 and condensed. The product was purified using flash chromatography (stationary phase: silica gel; eluent gradient: 0:10 (v:v) to 3:2 (v:v) of ethyl acetate and hexanes in 200 mL increments. The product was condensed to afford 0.96 g (72%) of **8** (white crystals). ^{1}H NMR (500 MHz, Chloroform-*d*) δ 8.06 (d, J = 9.1 Hz, 1H), 7.43 (t, J = 8.0 Hz, 2H), 7.30 – 7.23 (m, 1H), 7.10 – 7.04 (m, 2H), 6.99 (d, J = 2.6 Hz, 1H), 6.96 (dd, J = 9.1, 2.7 Hz, 1H), 5.55 (d, J = 9.1 Hz, 1H), 3.94 (dtd, J = 8.9, 7.6, 3.8 Hz, 1H), 1.64 (dqd, J = 14.7, 7.4, 5.4 Hz, 2H), 1.54 – 1.43 (m, 2H), 0.96 (t, J = 7.4 Hz, 6H). ^{13}C NMR (151 MHz, $CDCl_3$) δ 169.00, 162.29, 130.79, 129.90, 126.95, 126.12, 120.82, 119.42, 117.67, 115.24, 77.47, 77.26, 77.05. HRMS (ESI) m/z calculated for $C_{18}H_{20}N_2NaO_4$: [M+Na]$^+$ 351.1321, found 351.1067.

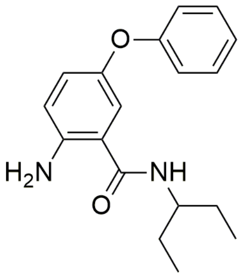

2-amino-N-(pentan-3-yl)-5-phenoxybenzamide (9) (Scheme S3). 8 (400 mg, 2.9 mmol), Pd/C (60 mg, 15% by weight), and 5mL of ethyl acetate was transferred in a dry, Ar purged 100 mL round bottom flask with a stir bar. The round bottom was then vacuumed for 5 min. H_2 gas was attached to the round bottom and the reaction was stirred overnight. The reaction was then vacuumed filtered and condensed giving crude **9** (black oil). HRMS (ESI) m/z calculated for $C_{18}H_{23}N_2O_2$: [M+H]$^+$ 299.1760, found 299.1658.

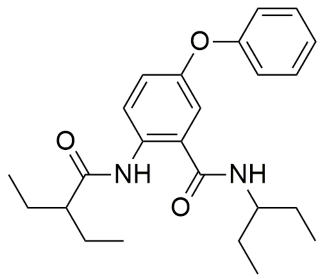

2-(2-ethylbutanamido)-N-(pentan-3-yl)-5-phenoxybenzamide (Fox) (Scheme S3). 2-ethylbutyric acid (460 µL, 3.6 mmol) was placed in a 100 mL round bottom flask with a stir bar, and purged with argon. The reagent was dissolved in DCM, with 3 drops of DMF. The reaction mixture was cooled to -78 °C and was stirred for 5 minutes. Then, (920 µL, 12 mmol) of oxalyl chloride was added dropwise, the stirred for 1 hr. The reaction was monitored via TLC by taking a few drops of reaction mixture and adding it to methanol. This would generate the ester from and move up the TLC plate. The reaction mixture was condensed 3 times, each time adding 20 mL of DCM. The condensed product was dissolved in 20 mL of DCM and cooled to -78°C for 5 min. **9** (from 1.2 mmol of **8**) was dissolved in DCM and added dropwise followed by NMM (2.00 mL, 19 mmol). This was raised to room temperature and reacted overnight. Upon completion of the reaction, the mixture was quenched with 100 mL of 5% HCl. The mixture was extracted with DCM (3×25 mL). The organic layer was dried Na_2SO_4 and condensed. The product was purified using flash chromatography (stationary phase: silica gel; eluent gradient: 0:10 (v:v) to 2:3 (v:v) of ethyl acetate and hexanes in 200 mL increments. The product was condensed to afford 117 mg (25%) of **Fox** (white crystals). ^{1}H NMR (500 MHz, Chloroform-*d*) δ 8.06 (d, J = 9.0 Hz, 1H), 7.47 – 7.40 (m, 2H), 7.27 (td, J = 7.4, 1.2 Hz, 1H), 7.10 – 7.04 (m, 2H), 6.99 (d, J = 2.6 Hz, 1H), 6.96 (dd, J = 9.1, 2.7 Hz, 1H), 5.55 (d, J = 9.1 Hz, 1H), 3.94 (dtd, J = 8.9, 7.6, 3.8 Hz, 1H), 1.63 (dtd, J = 14.8, 7.4, 5.5 Hz, 2H), 1.54 – 1.43 (m, 2H), 0.96 (t, J = 7.4 Hz, 6H). ^{13}C NMR (151 MHz, $CDCl_3$) δ 180.66, 175.05, 168.39, 160.00, 157.58, 151.74, 135.06, 130.04, 123.76, 123.47, 123.40, 123.04, 118.19, 117.43, 77.44, 77.23, 77.02, 53.22, 52.95, 52.91, 48.71, 27.68, 27.38, 25.96, 24.99, 12.18, 11.94, 10.48, 10.41. HRMS (ESI) m/z calculated for $C_{24}H_{32}N_2NaO_3$: $[M+Na]^+$ 419.2311, found 419.2108

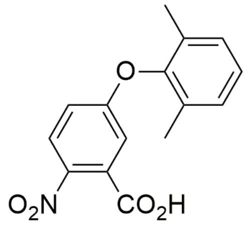

5-(2,6-dimethylphenoxy)-2-nitrobenzoic acid (5b) (Scheme S4). 4 (1.9 g, 10 mmol), was placed in in a pressure tube bottom flask with a stir bar, and purged with argon. 20 mL of dry toluene was added, followed by the cesium carbonate (4.9 g, 57 mmol), and 2,6-dimethylphenol (2.4 g, 20 mmol). The mixture was stirred at 110 °C overnight. The progress of the reaction was monitored via TLC. The reaction was taken out of the oil bath for 10 minutes to cool and then diluted with 200 mL of 5% HCl and extracted with DCM (3×25 mL). Dried over Na_2SO_4 and vacuum filtered. The filtrate was condensed. The product was crashed out in DCM and hexanes at room temperature. The product was condensed to afford 1.21 g of (42%) of **5b** (light yellow crystals). ^{1}H NMR (500 MHz, Chloroform-*d*) δ 7.98 (d, J = 9.0 Hz, 1H), 7.13 (d, J = 16.6 Hz, 4H), 6.92 (dd, J = 9.0, 2.6 Hz, 1H), 2.12 (s, 6H). ^{13}C NMR (126 MHz, $cdcl_3$) δ 171.02, 161.70, 150.04, 141.25, 130.91, 130.31, 129.76, 127.20, 126.58, 116.96, 115.68, 77.51, 77.26, 77.00, 16.39. HRMS (ESI) m/z calculated for $C_{15}H_{12}NO_5$: $[M-H]^-$ 286.0721, found 286.0756.

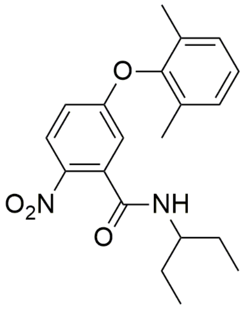

5-(2,6-dimethylphenoxy)-2-nitro-N-(pentan-3-yl)benzamide (10) (Scheme S4). 5b (9.9 g, 34 mmol) was transferred to a dry, Ar purged 150 mL round bottom flask, with a stir bar. This was dissolved in 40 mL of DCM, with 3 drops of DMF. The reaction mixture was cooled to -78°C and was stirred for 5 minutes. Then, (6.9 mL, 80 mmol) of oxalyl chloride was added dropwise, the stirred for 1 hr. The reaction was monitored via TLC by taking a few drops of reaction mixture and adding it to methanol. This would generate the ester from and move up the TLC plate. The reaction mixture was condensed 3 times, each time adding 20 mL of DCM. The condensed product was dissolved in 20 mL of DCM and cooled to -78°C for 5 min. 3-aminopentane (4.7 mL, 40 mmol) was added dropwise followed by NMM (11 mL, 100 mmol). This was raised to room temperature and reacted overnight. Upon completion of the reaction, the mixture was quenched with 200 mL of 5% HCl. The mixture was extracted with DCM (3×50mL). The organic layer was dried Na_2SO_4 and condensed. The product was purified using flash chromatography (stationary phase: silica gel; eluent gradient: 0:10 (v:v) to 3:2 (v:v) of ethyl acetate and hexanes in 400 mL increments. The product was condensed to afford 3.19 g 26% of **10** (light yellow powder). ^{1}H NMR (600 MHz, Chloroform-*d*) δ 13.21 – 13.15 (m, 1H), 12.28 (d, *J* = 4.6 Hz, 3H), 12.10 (dd, *J* = 4.7, 2.6 Hz, 1H), 11.84 (ddt, *J* = 6.7, 4.6, 2.7 Hz, 1H), 10.81 (dd, *J* = 9.4, 4.9 Hz, 1H), 9.11 (h, *J* = 7.2, 5.1 Hz, 1H), 7.28 – 7.23 (m, 6H), 6.85 – 6.76 (m, 2H), 6.65 (dtt, *J* = 14.4, 7.5, 3.8 Hz, 2H), 6.13 (tt, *J* = 7.5, 3.7 Hz, 6H). ^{13}C NMR (126 MHz, cdcl$_3$) δ 166.30, 163.03, 149.94, 136.48, 136.13, 132.85, 130.25, 128.52, 127.52, 121.07, 116.28, 115.95, 77.49, 77.23, 76.98, 53.11, 27.28, 21.03, 16.14, 10.35. HRMS (ESI) m/z calculated for $C_{20}H_{24}N_2NaO_4$: $[M+Na]^+$ 379.1628, found 379.1608.

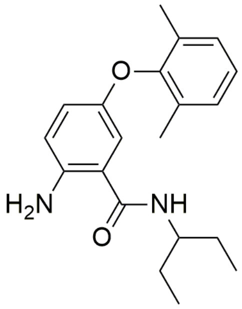

2-amino-5-(2,6-dimethylphenoxy)-N-(pentan-3-yl)benzamide (11) (Scheme S4). 10 (3.2 g, 9.0 mmol), Pd/C (1.6 g, 15% by weight), and 15 mL of ethyl acetate was transferred in a dry, Ar purged 100 mL round bottom flask with a stir bar. The round bottom was then vacuumed for 5 min. H_2 gas was attached to the round bottom and the reaction was stirred overnight. The reaction was then vacuumed filtered and condensed giving crude **11** (black oil). HRMS (ESI) m/z calculated for $C_{20}H_{24}N_2NaO_2$: $[M+Na–2H]^+$ 347.1735, found 347.0353.

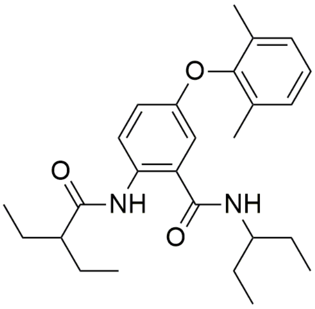

5-(2,6-dimethylphenoxy)-2-(2-ethylbutanamido)-N-(pentan-3-yl)benzamide (Dox) (Scheme S4, Chart 1d). 2-ethylbutyric acid (2.3 mL, 18 mmol) was placed in a 100 mL round bottom flask with a stir bar, and purged with argon. The reagent was dissolved in DCM, with 3 drops of DMF. The reaction mixture was cooled to -78°C and was stirred for 5 minutes. Then, (3.0 mL, 36 mmol) of oxalyl chloride was added dropwise, the stirred for 1 hr. The reaction was monitored via TLC by taking a few drops of reaction mixture and adding it to methanol. This would generate the ester from and move up the TLC plate. The reaction mixture was condensed 3 times, each time adding 20 mL of DCM. The condensed product was dissolved in 20 mL of DCM and cooled to -78°C for 5 min. **11** (from 9.0 mmol of **10**) was dissolved in DCM and added dropwise followed by NMM (5.0 mL, 45 mmol). This was raised to room temperature and reacted overnight. Upon completion of the reaction, the mixture was quenched with 100 mL of 5% HCl. The mixture was extracted with DCM (3×25mL). The organic layer was dried Na_2SO_4 and condensed. The product was purified using flash chromatography (stationary phase: silica gel; eluent gradient: 0:10 (v:v) to 3:2 (v:v) of ethyl acetate and hexanes in 200 mL increments. The product was condensed to afford 1.1 g (29%) of **Dox** (white crystals). ^{1}H NMR (600 MHz, Chloroform-*d*) δ 10.49 (s, 1H), 8.36 (d, J = 9.1 Hz, 1H), 7.10 (d, J = 6.3 Hz, 1H), 7.09 – 7.05 (m, 1H), 7.04 (d, J = 2.8 Hz, 1H), 6.59 (dd, J = 9.1, 2.8 Hz, 1H), 5.81 (d, J = 9.1 Hz, 1H), 3.95 (dt, J = 9.0, 5.2 Hz, 1H), 2.11 (s, 6H), 2.07 (dt, J = 9.3, 5.1 Hz, 1H), 1.72 – 1.61 (m, 5H), 1.57 – 1.51 (m, 2H), 1.51 – 1.43 (m, 2H), 0.93 (dt, J = 14.8, 7.4 Hz, 12H). ^{13}C NMR (151 MHz, $CDCl_3$) δ 174.80, 168.65, 153.24, 150.86, 132.93, 131.52, 129.38, 125.67, 123.98, 123.84, 116.91, 113.07, 77.44, 77.23, 77.02, 52.91, 27.73, 26.03, 16.56, 12.24, 10.50. HRMS (ESI) m/z calculated for $C_{26}H_{35}N_2O_3$: $[M-H]^-$ 423.5667, found 423.2622.

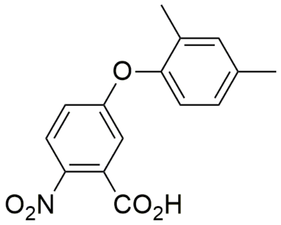

5-(2,4-dimethylphenoxy)-2-nitrobenzoic acid (5c) (Scheme S5). 4 (1.9 g, 10 mmol), was placed in in a pressure tube bottom flask with a stir bar, and purged with argon. 20 mL of dry toluene was added, followed by the cesium carbonate (4.9 g, 57 mmol), and 2,4-Dimethylphenol (2.4 mL, 20 mmol). The mixture was stirred at 110°C overnight. The progress of the reaction was monitored via TLC. The reaction was taken out of the oil bath for 10 minutes to cool and then diluted with 200 mL of 5% HCl and extracted with DCM (3×25mL). Dried over Na_2SO_4 and vacuum filtered. The filtrate was condensed. The product was crashed out in DCM and hexanes at room temperature. The product was purified using flash chromatography (stationary phase: silica gel; eluent gradient: 0:10 (v:v) to 6:4 (v:v) of ethyl acetate and hexanes in 200 mL increments. At the 1:1 (v:v) gradient, 1% of acetic acid was added to the elution. The product was condensed to afford 2.36 g of (88%) of **5c** (tan powder). ^{1}H NMR (500 MHz, Chloroform-*d*) δ 7.98 (d, J = 9.0 Hz, 1H), 7.12 (t, J = 2.9 Hz, 2H), 7.07 (dd, J = 8.2, 2.2 Hz, 1H), 7.01 (dd, J = 9.0, 2.7 Hz, 1H), 6.90 (d, J = 8.1 Hz, 1H), 2.36 (s, 3H), 2.13 (s, 3H). ^{13}C NMR (126 MHz, $cdcl_3$) δ 170.39, 162.59, 149.89, 141.27, 136.26, 132.96, 130.24, 130.02, 128.67, 127.01, 121.12, 118.14, 116.38, 77.49, 77.23, 76.98, 21.06, 16.14. HRMS (ESI) m/z calculated for $C_{15}H_{12}NO_5$: $[M–H]^-$ 286.0721, found 286.0722.

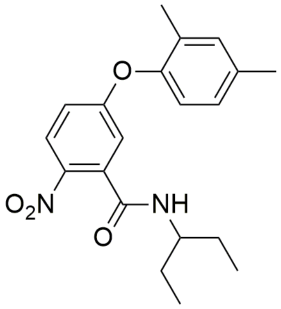

5-(2,4-dimethylphenoxy)-2-nitro-N-(pentan-3-yl)benzamide (12) (Scheme S5). 5c (2.0 g, 7.5 mmol) was transferred to a dry, Ar purged 100 mL round bottom flask, with a stir bar. The material was

dissolved in 20 mL of DCM, with 3 drops of DMF. The reaction mixture was cooled to -78°C and was stirred for 5 minutes. 2.5 mL (30 mmol) of oxalyl chloride was added dropwise, and stirred for 1 hr The reaction was monitored using TLC by taking a few drops of reaction mixture and adding it to methanol. This would generate the ester from and move up the TLC plate. The reaction mixture was condensed 3 times, each time adding 20 mL of DCM. The condensed product was dissolved in 20 mL of DCM and cooled to -78°C for 5 min. 3-aminopentane (1.8 mL, 15 mmol) was added dropwise followed by NMM (4.1 mL, 38 mmol). This was raised to room temperature and reacted overnight. Upon completion of the reaction, the mixture was quenched with 200 mL of 5% HCl. The mixture was extracted with DCM (3×50mL). The organic layer was dried Na_2SO_4 and condensed. The product was purified using flash chromatography (stationary phase: silica gel; eluent gradient: 0:10 (v:v) to 3:2 (v:v) of ethyl acetate and hexanes in 400 mL increments. The product was condensed to afford 1.83 g (64%) of **12** (light yellow crystals). ^{1}H NMR (500 MHz, Chloroform-*d*) δ 8.04 (d, *J* = 9.1 Hz, 1H), 7.11 (d, *J* = 2.2 Hz, 1H), 7.04 (dd, *J* = 8.2, 2.2 Hz, 1H), 6.96 (d, *J* = 2.7 Hz, 1H), 6.86 (d, *J* = 8.1 Hz, 1H), 6.80 (dd, *J* = 9.1, 2.7 Hz, 1H), 5.46 (d, *J* = 9.1 Hz, 1H), 3.97 (dtt, *J* = 9.0, 7.6, 5.5 Hz, 1H), 2.35 (s, 3H), 2.12 (s, 3H), 1.65 (dddd, *J* = 14.9, 13.0, 7.5, 5.5 Hz, 2H), 1.56 – 1.44 (m, 2H), 0.98 (t, *J* = 7.4 Hz, 6H). ^{13}C NMR (151 MHz, $CDCl_3$) δ 166.31, 163.02, 149.93, 139.74, 136.47, 136.12, 132.84, 130.24, 128.52, 127.50, 121.06, 116.28, 115.94, 77.44, 77.23, 77.02, 53.10, 27.26, 21.02, 16.13, 10.34. HRMS (ESI) m/z calculated for $C_{20}H_{24}N_2NaO_4$: $[M+Na]^+$ 379.1634, found 379.0181.

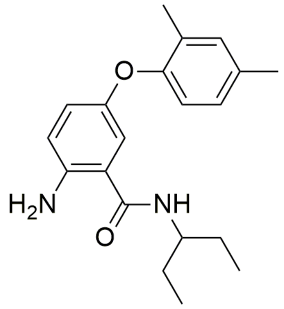

2-amino-5-(2,4-dimethylphenoxy)-N-(pentan-3-yl)benzamide (13) (Scheme S5). **12** (2.4 g, 6.8 mmol), Pd/C (360 mg, 15% by weight), and 15 mL of ethyl acetate was transferred in a dry, Ar purged 100 mL round bottom flask with a stir bar. The round bottom was then vacuumed for 5 min. H_2 gas was attached to the round bottom and the reaction was stirred overnight. The reaction was then vacuumed filtered and condensed giving crude **13** (black oil). HRMS (ESI) m/z calculated for $C_{20}H_{26}N_2NaO_2$: $[M+Na]^+$ 349.1892, found 348.9783.

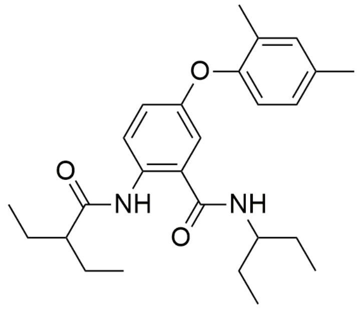

5-(2,4-dimethylphenoxy)-2-(2-ethylbutanamido)-N-(pentan-3-yl)benzamide (Dox_{24}) (Scheme S5). HATU (3.6 g, 9.47 mmol), and HOAt (1.4 g, 9.47 mmol), 2-ethylbutyric acid (1.2 mL, 9.47 mmol), DIPEA (1.2 mL, 9.47 mmol) was dissolved in 20 mL of DMF in a dry, argon purged 100 mL round bottom flask with a stir bar. This was stirred for 15 minutes and then **13** (from 6.8 mmol of **12**) was transferred and stirred overnight. The progress of the reaction was monitored via TLC. The mixture quenched with 100 mL saturated Na_2CO_3 was extracted with ethyl acetate (3×50mL). The organic layer was dried Na_2SO_4 and condensed. The product was purified using flash chromatography (stationary phase: silica gel; eluent gradient: 0:10 (v:v) to 1: (v:v) of ethyl acetate and hexanes in 400 mL increments.to afford 1.66 g (58%) of **Dox_{24}** (white crystals). ^{1}H NMR (500 MHz, Chloroform-*d*) δ 10.61 (s, 1H), 8.46 (d, *J* = 9.1 Hz, 1H), 7.08 (dd, *J* = 9.7, 2.5 Hz, 2H), 6.96 (dd, *J* = 8.1, 2.2 Hz, 1H), 6.87 (dd,

J = 9.1, 2.8 Hz, 1H), 6.74 (d, *J* = 8.2 Hz, 1H), 5.77 (d, *J* = 9.2 Hz, 1H), 3.95 (ddt, *J* = 13.5, 8.3, 4.3 Hz, 1H), 2.31 (s, 3H), 2.19 (s, 3H), 2.11 (tt, *J* = 9.0, 5.3 Hz, 1H), 1.75 – 1.61 (m, 4H), 1.60 – 1.51 (m, 2H), 1.46 (dt, *J* = 13.8, 7.5 Hz, 2H), 0.94 (td, *J* = 7.4, 4.2 Hz, 12H). ^{13}C NMR (126 MHz, cdcl$_3$) δ 174.89, 168.54, 153.16, 152.28, 133.98, 132.43, 129.50, 127.93, 123.72, 123.49, 120.50, 119.20, 115.53, 77.49, 77.23, 76.98, 52.92, 27.73, 26.01, 20.91, 16.30, 12.23, 10.49. HRMS (ESI) m/z calculated for $C_{26}H_{35}N_2O_3$: $[M\text{-}H]^-$ 423.2653, found 423.2646.

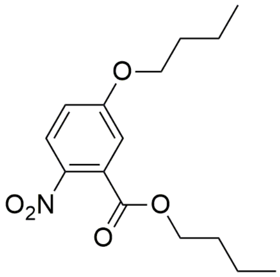

butyl 5-butoxy-2-nitrobenzoate (2a). (*a side product from pressure-tube conventional-heating synthesis of* **3a**) **1** (1.8 g, 10 mmol), was placed in in a pressure tube bottom flask with a stir bar, and purged with argon. Fifteen mL of 2ME was added, followed by the cesium carbonate (4.89 g, 15 mmol), and 1-iodobutane (2.3 mL, 20 mmol). The mixture was stirred at 130°C overnight. The progress of the reaction was monitored via TLC. The reaction was taken out of the oil bath, allowed to cool and diluted with 200 mL of 5% HCl and extracted with DCM (3×25mL). Dried over Na_2SO_4 and vacuum filtered. The filtrate was condensed. The product was purified using flash chromatography (stationary phase: silica gel; eluent gradient: 0:10 (v:v) to 1:1 (v:v) of ethyl acetate and hexanes in 200 mL increments. The product was condensed to afford 10 mg (0.3%) of **2a** (brown oil). ^{1}H NMR (500 MHz, Chloroform-*d*) δ 7.98 (d, *J* = 9.0 Hz, 1H), 7.01 (d, *J* = 2.7 Hz, 1H), 6.97 (dd, *J* = 9.0, 2.7 Hz, 1H), 4.31 (t, *J* = 6.7 Hz, 2H), 4.04 (t, *J* = 6.4 Hz, 2H), 1.82 – 1.64 (m, 4H), 1.53 – 1.34 (m, 4H), 0.94 (dt, *J* = 15.0, 7.4 Hz, 6H). HRMS (ESI) m/z calculated for $C_{15}H_{20}NO_5$: $[M\text{-}H]^-$ 294.1347, found 294.3283.

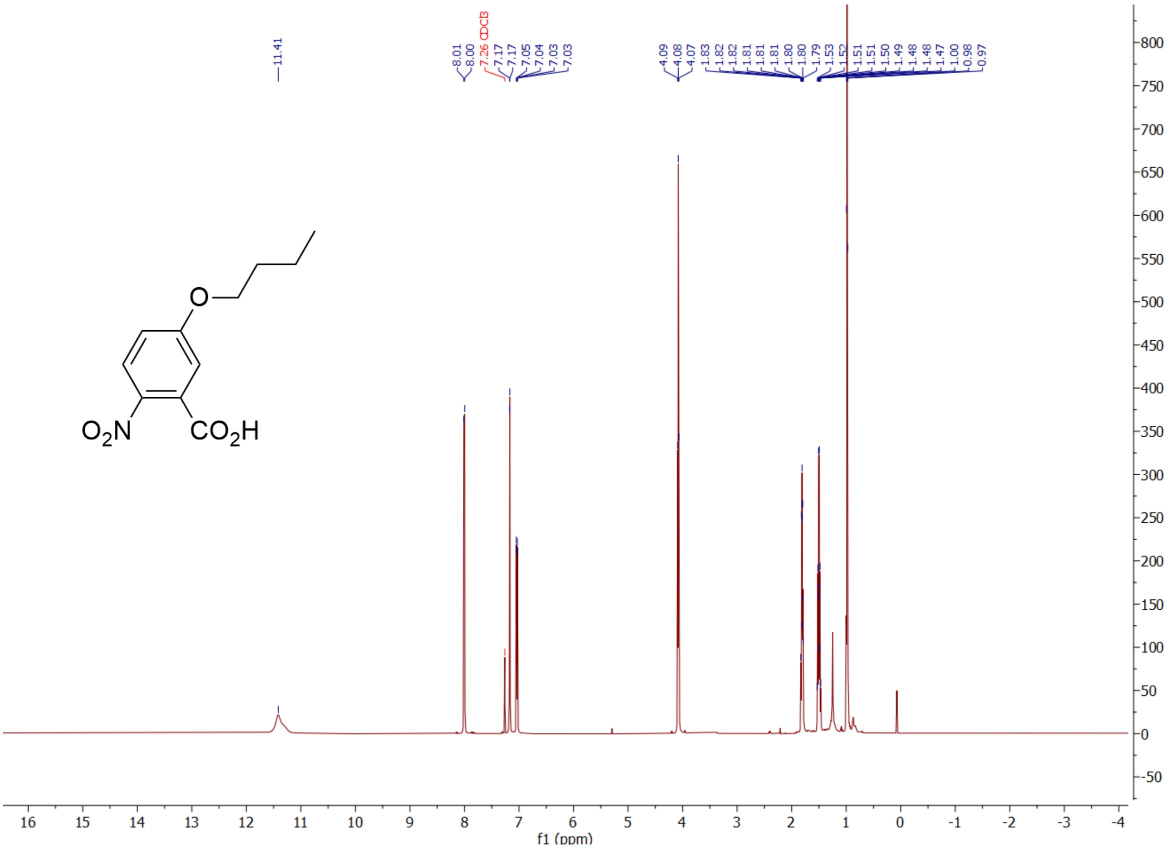

a

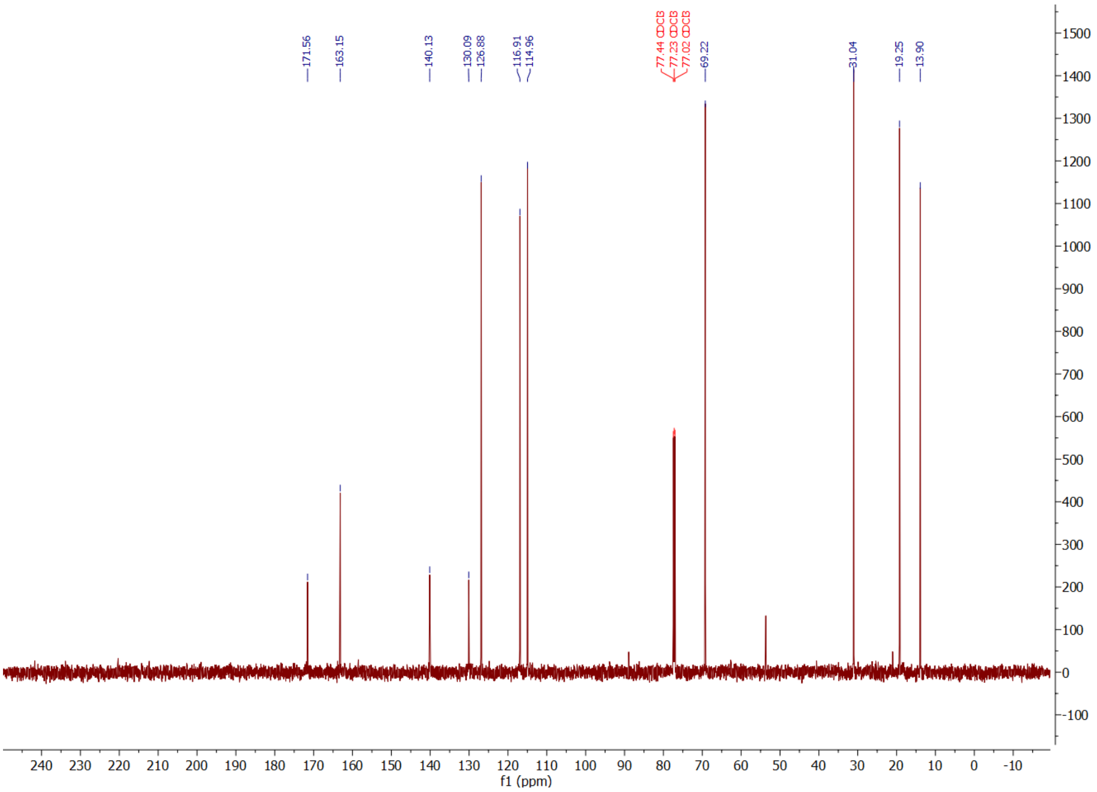

b

Figure S1. (a) ^{1}H NMR of **(3a)** (600 MHz, $CDCl_3$); (b) ^{13}C NMR of **(3a)** (151 MHz, $CDCl_3$).

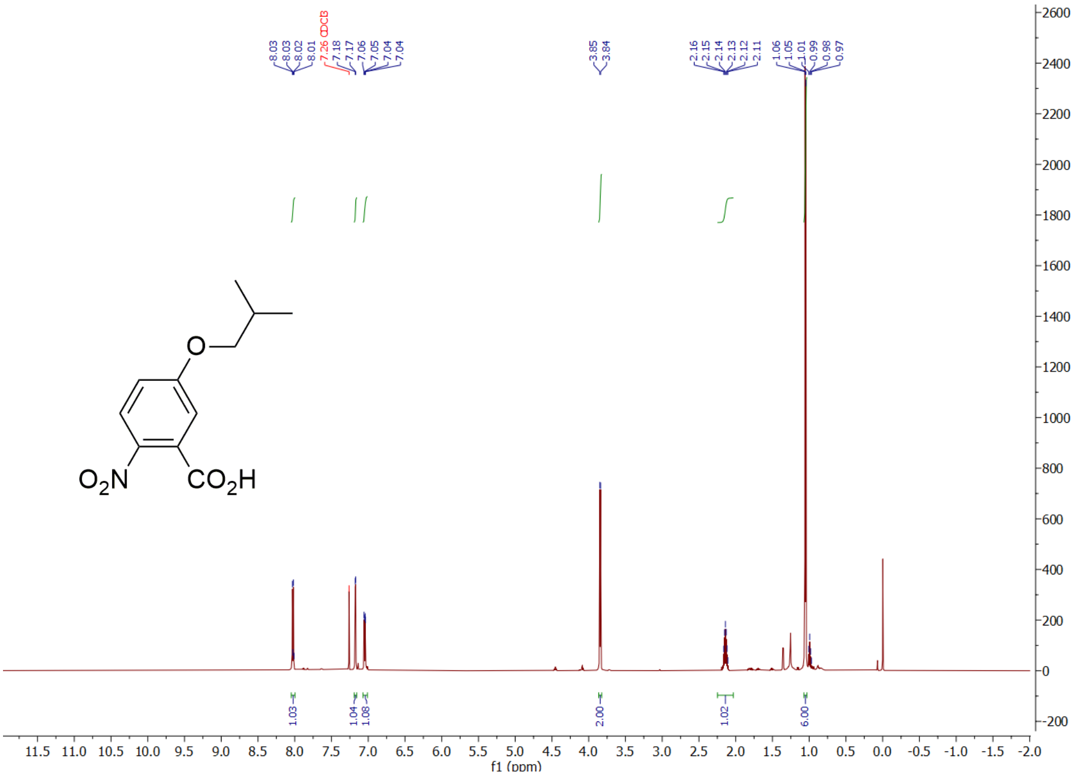

a

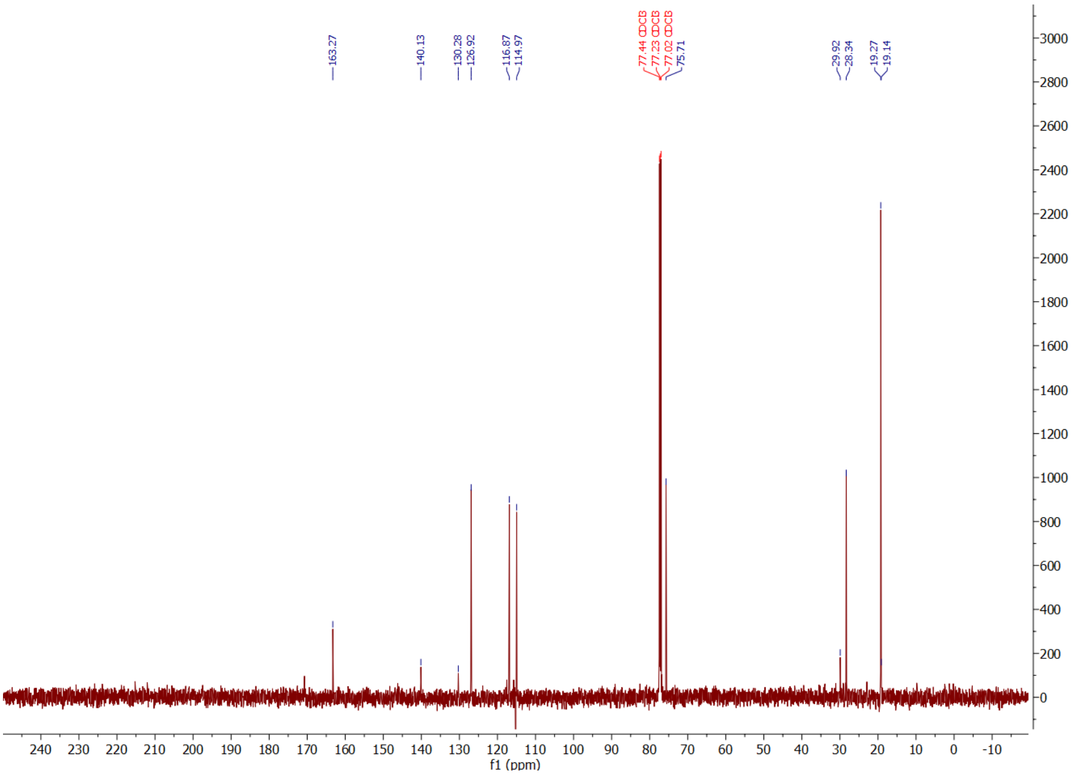

b

Figure S2. (a) ^{1}H NMR of **(3b)** (600 MHz, $CDCl_3$); (b) ^{13}C NMR of **(3b)** (151 MHz, $CDCl_3$).

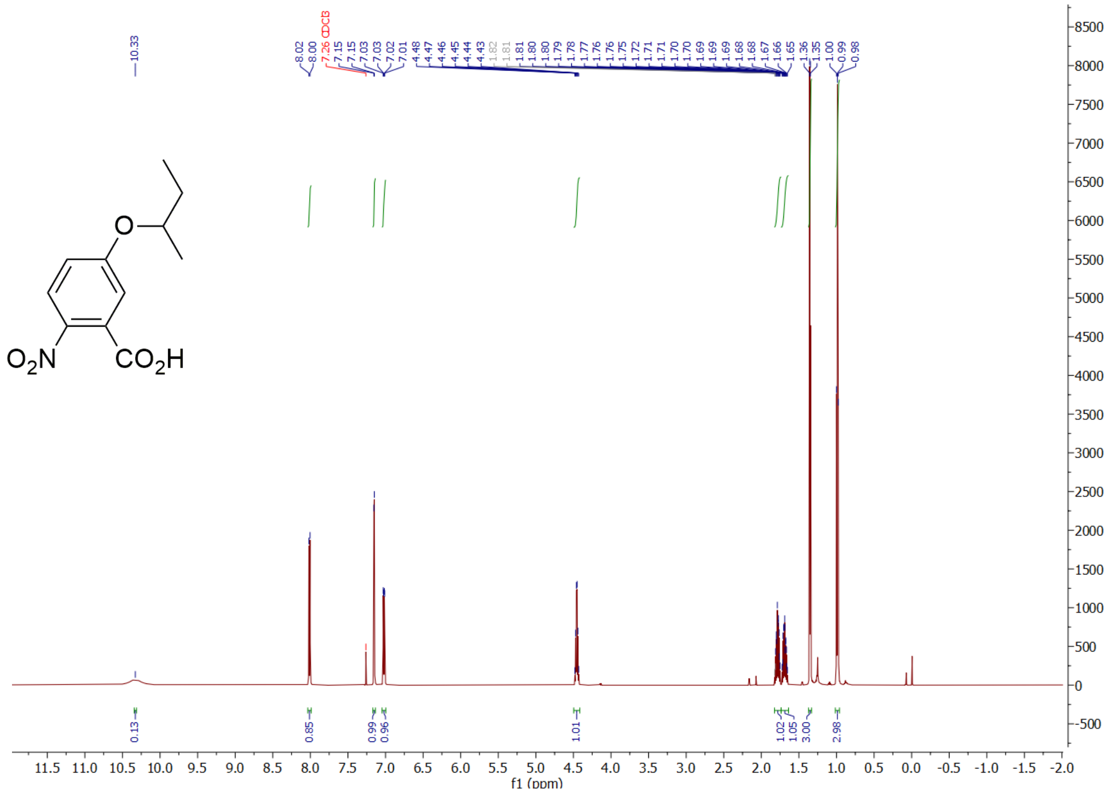

a

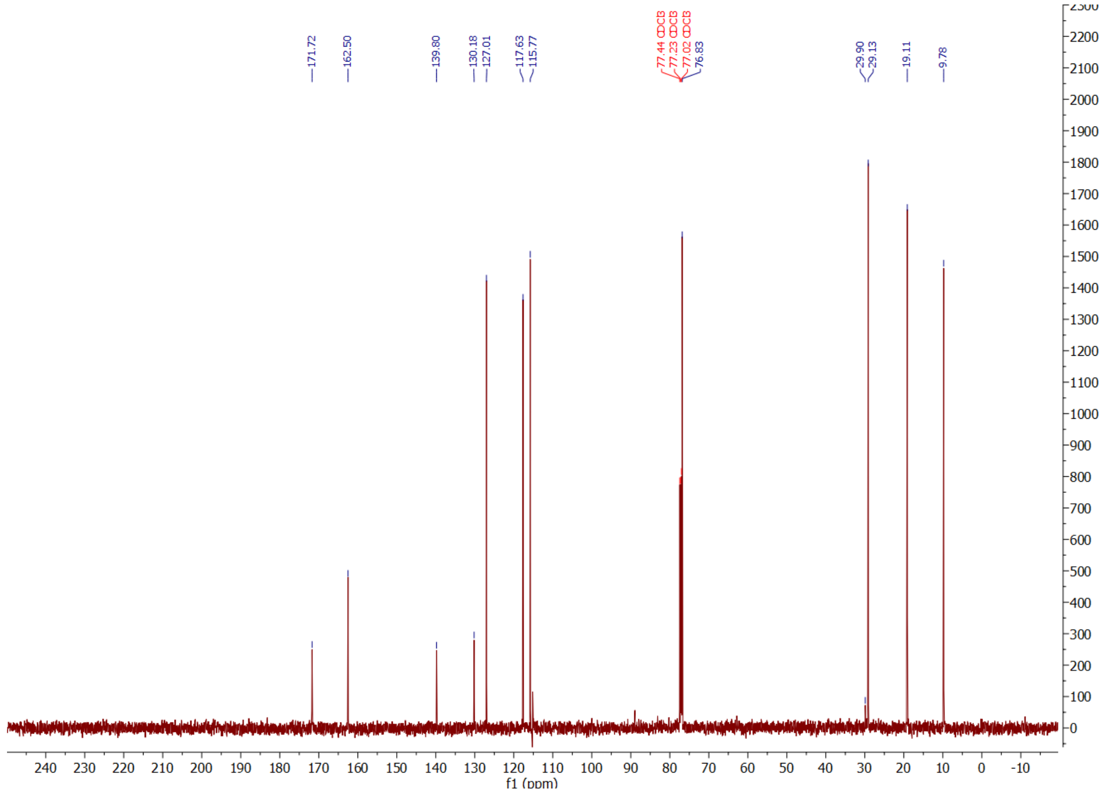

b

Figure S3. (a) ^{1}H NMR of **(3c)** (600 MHz, $CDCl_3$); (b) ^{13}C NMR of **(3c)** (151 MHz, $CDCl_3$).

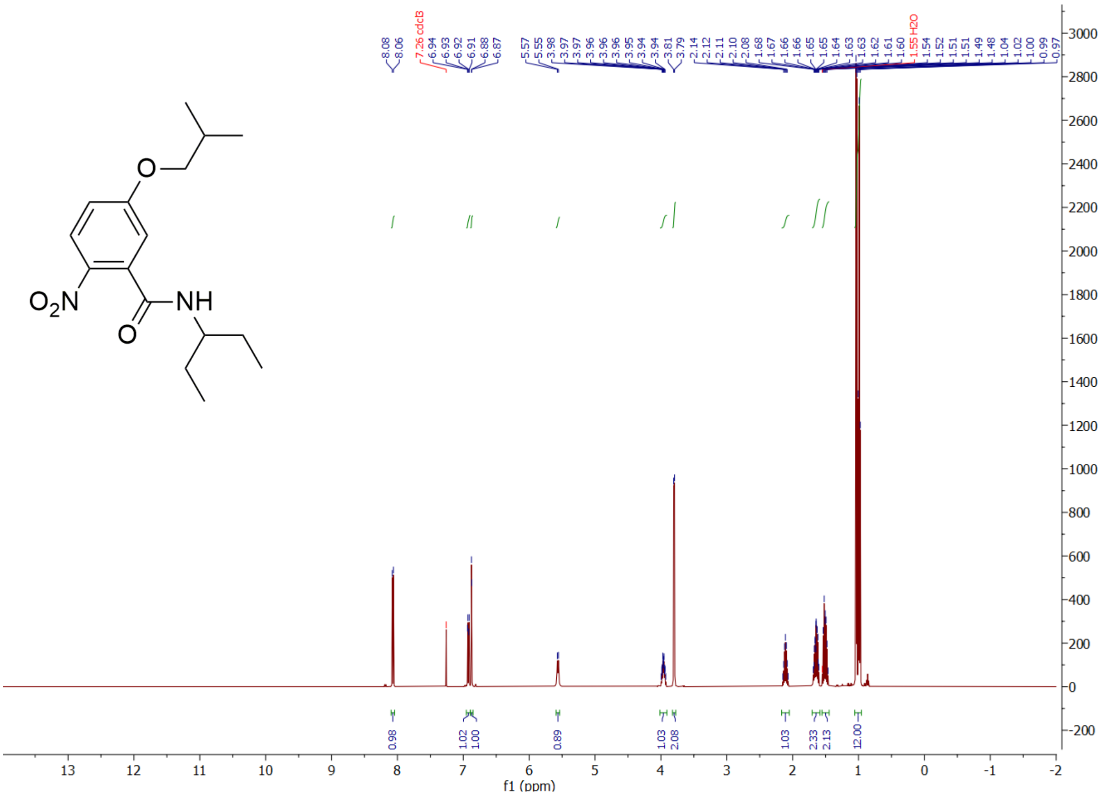

a

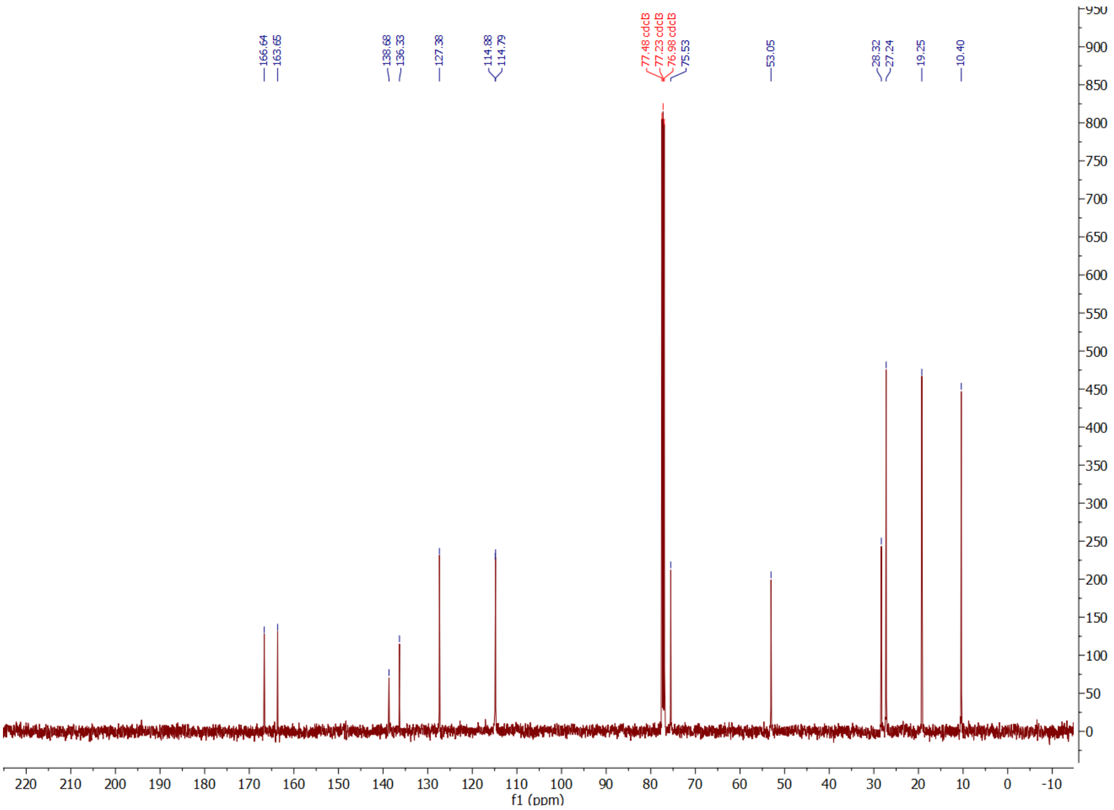

b

Figure S4. (a) ^{1}H NMR of **(6)** (500 MHz, $CDCl_3$); (b) ^{13}C NMR of **(6)** (126 MHz, $CDCl_3$).

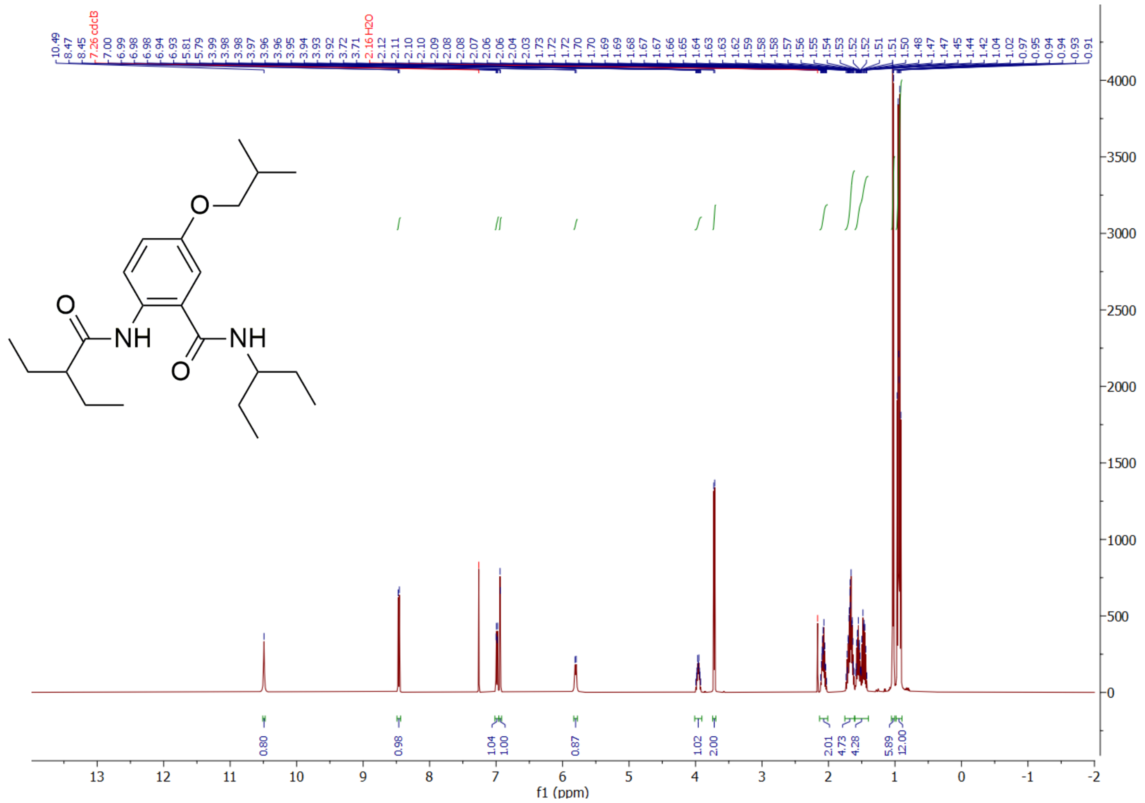

a

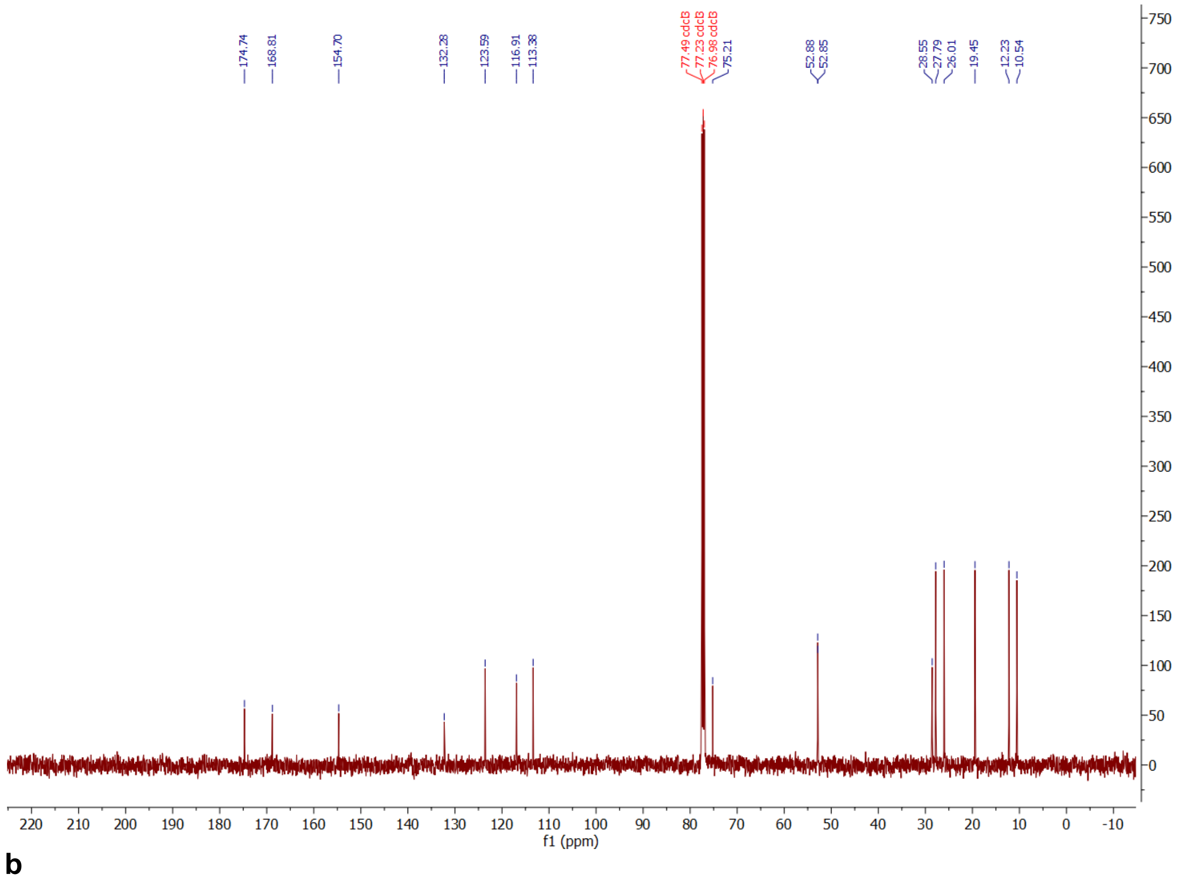

b

Figure S5. (a) ^{1}H NMR of **(Box)** (500 MHz, $CDCl_3$); (b) ^{13}C NMR of **(Box)** (126 MHz, $CDCl_3$).

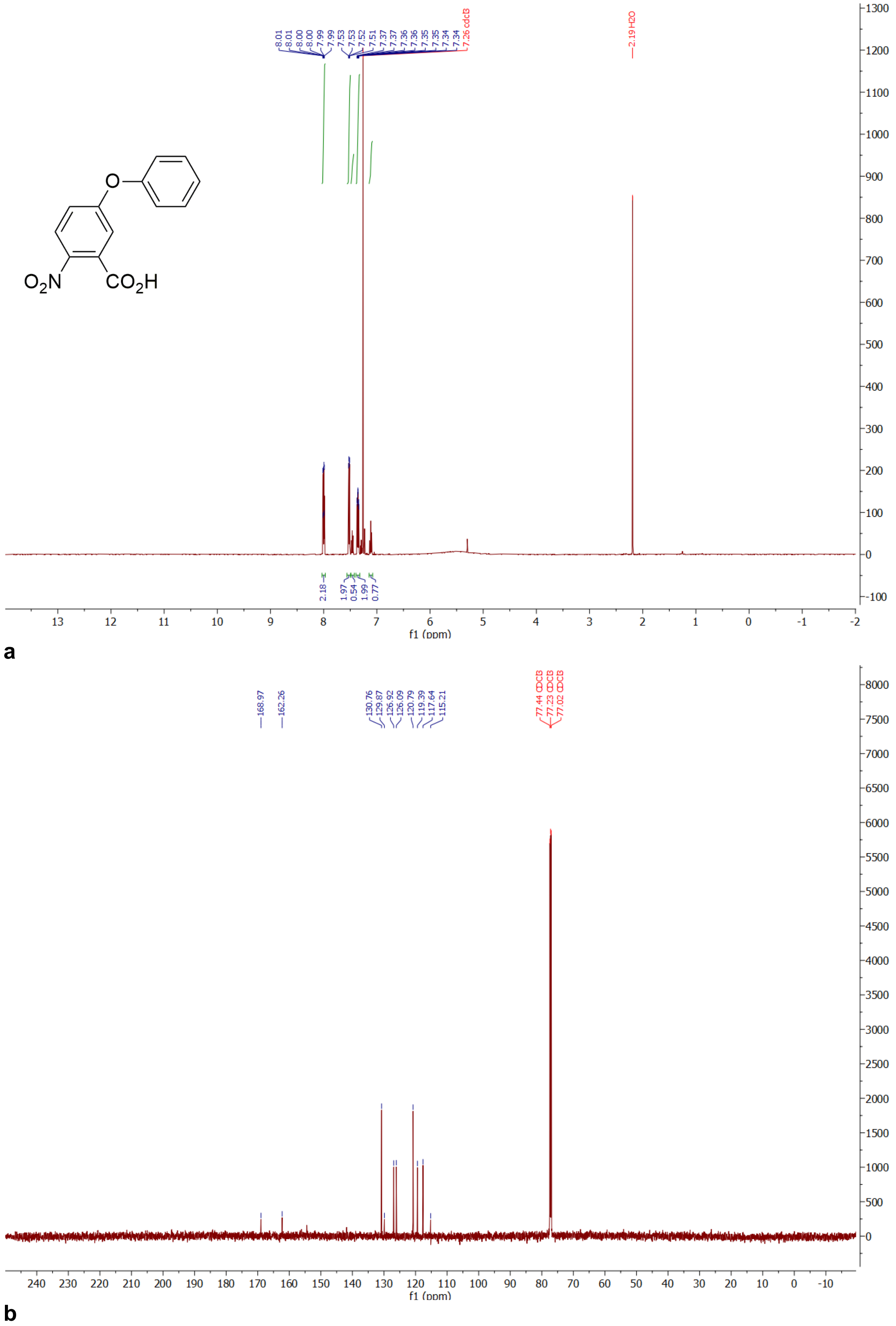

Figure S6. (a) ^{1}H NMR of **(5a)** (500 MHz, $CDCl_3$); (b) ^{13}C NMR of **(5a)** (151 MHz, $CDCl_3$).

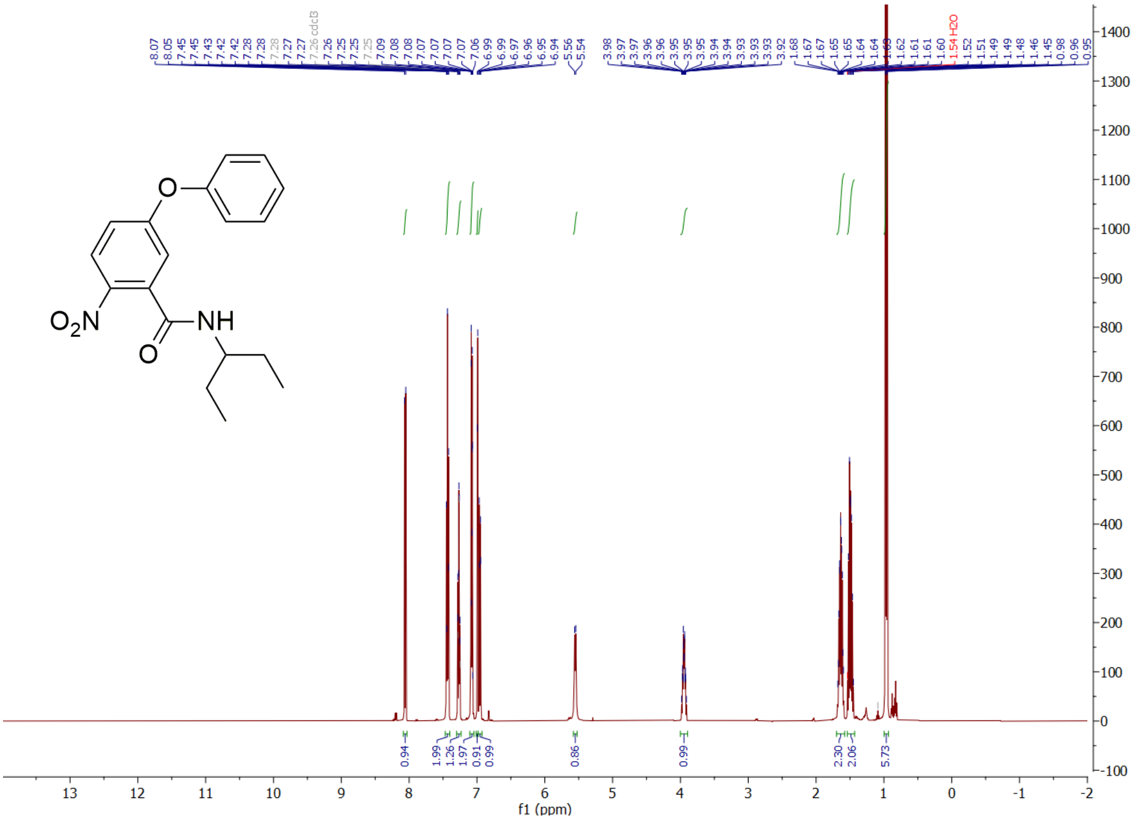

a

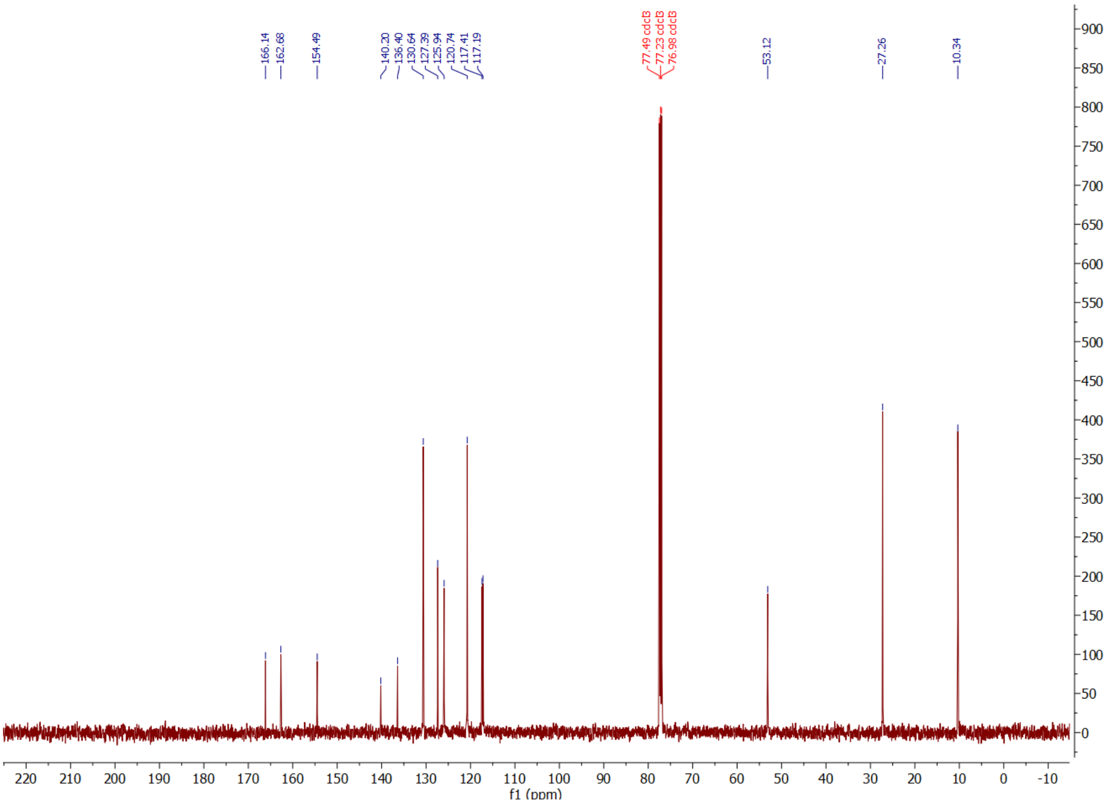

b

Figure S7. (a) ^{1}H NMR of **(8)** (500 MHz, $CDCl_3$); (b) ^{13}C NMR of **(8)** (151 MHz, $CDCl_3$).

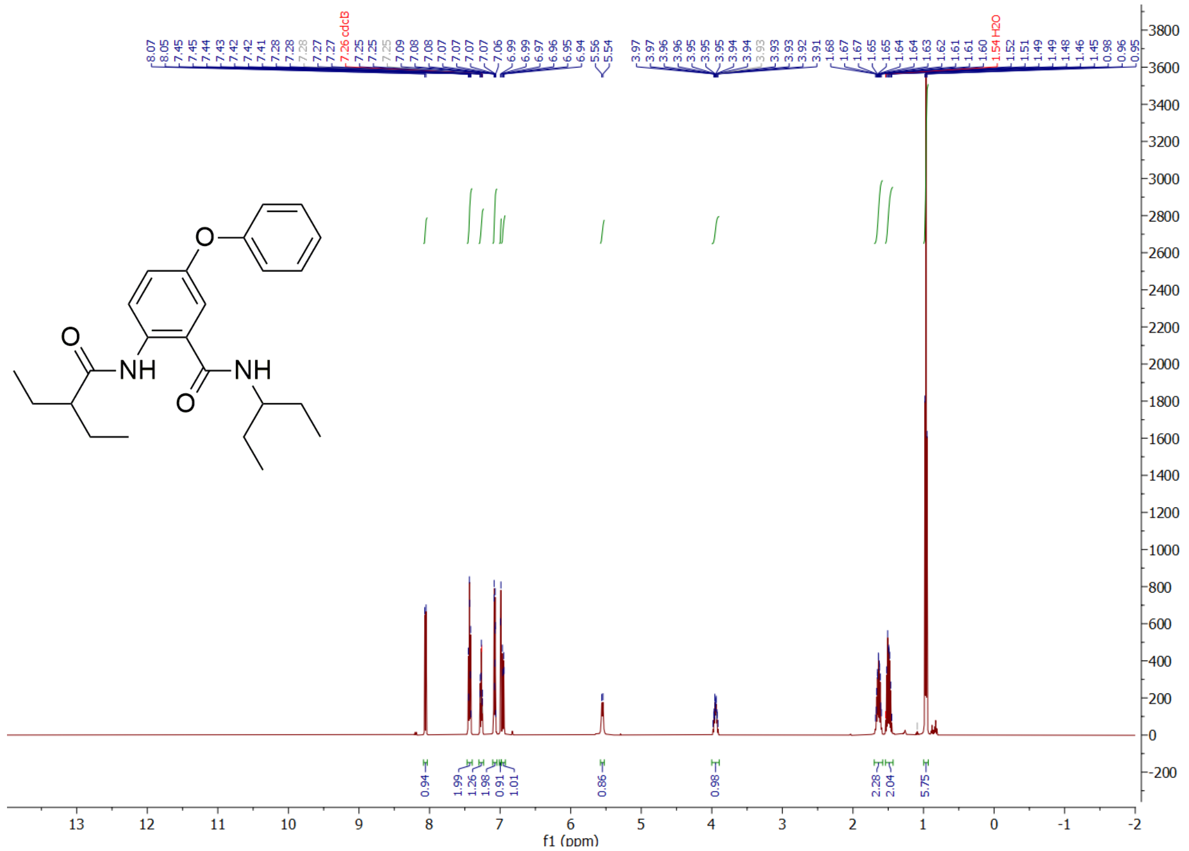

a

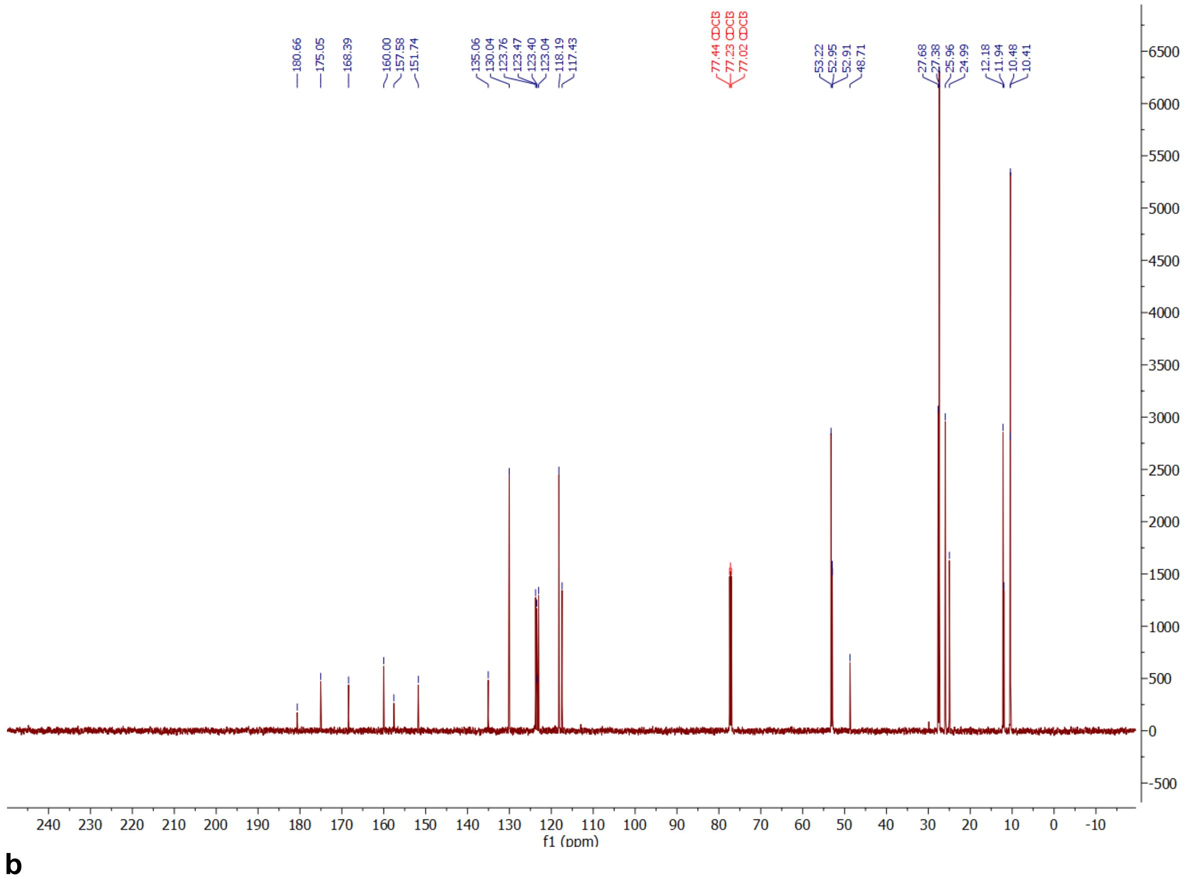

b

Figure S8. (a) ^{1}H NMR of **(Fox)** (600 MHz, $CDCl_3$); (b) ^{13}C NMR of **Fox** (126 MHz, $CDCl_3$).

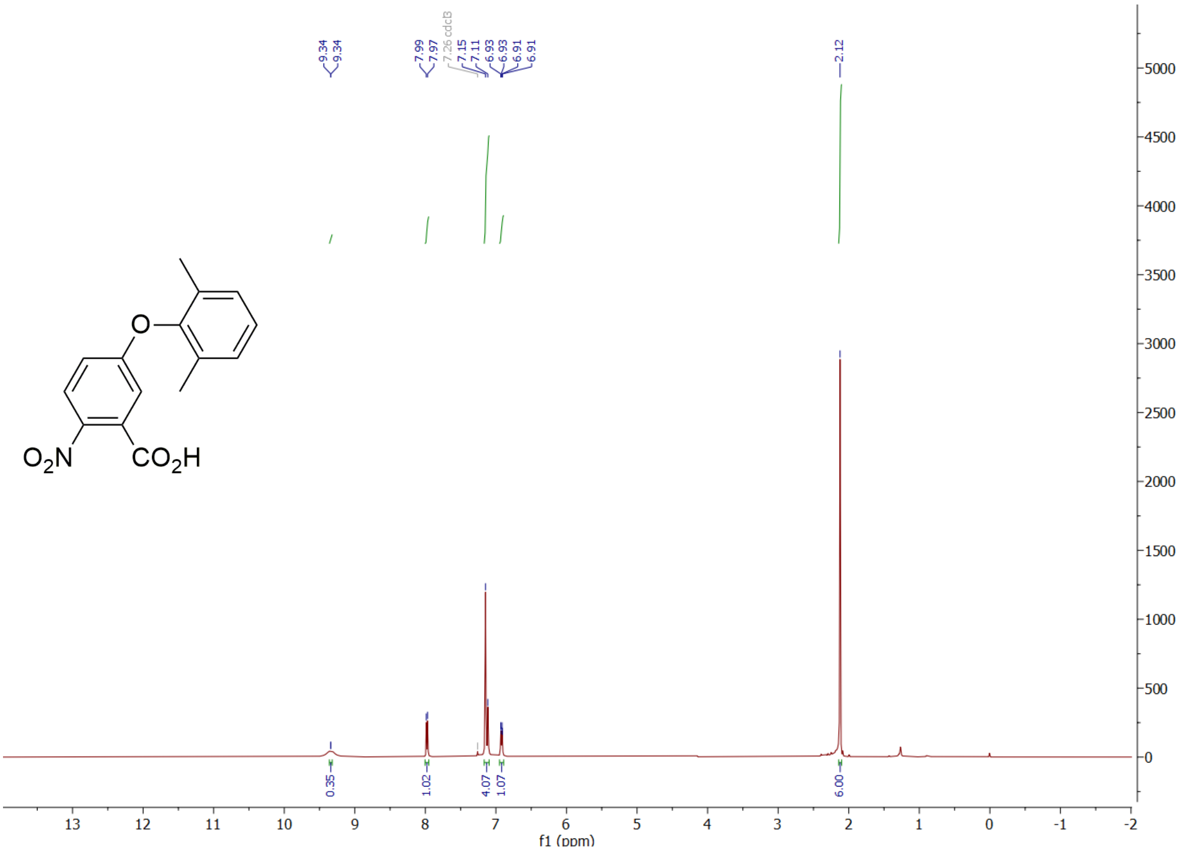

a

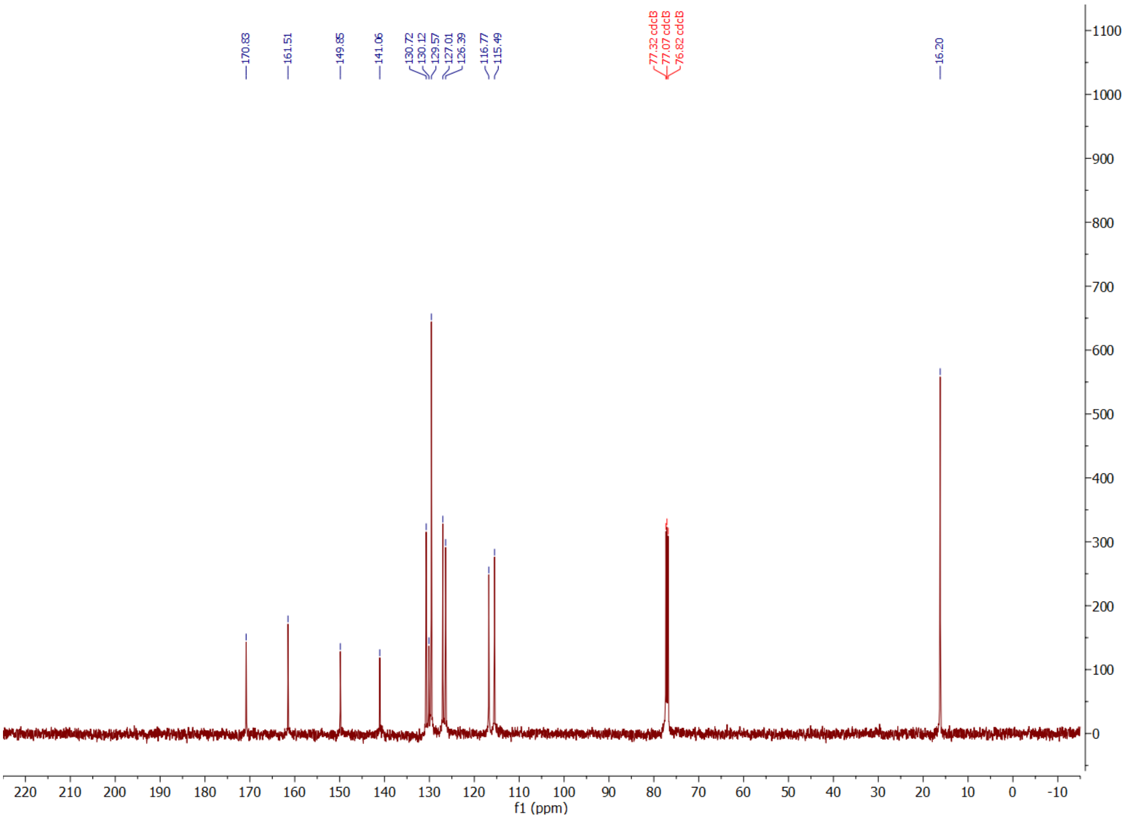

b

Figure S9. (a) ^{1}H NMR of **(5b)** (500 MHz, $CDCl_3$); (b) ^{13}C NMR of **(5b)** (126 MHz, $CDCl_3$).

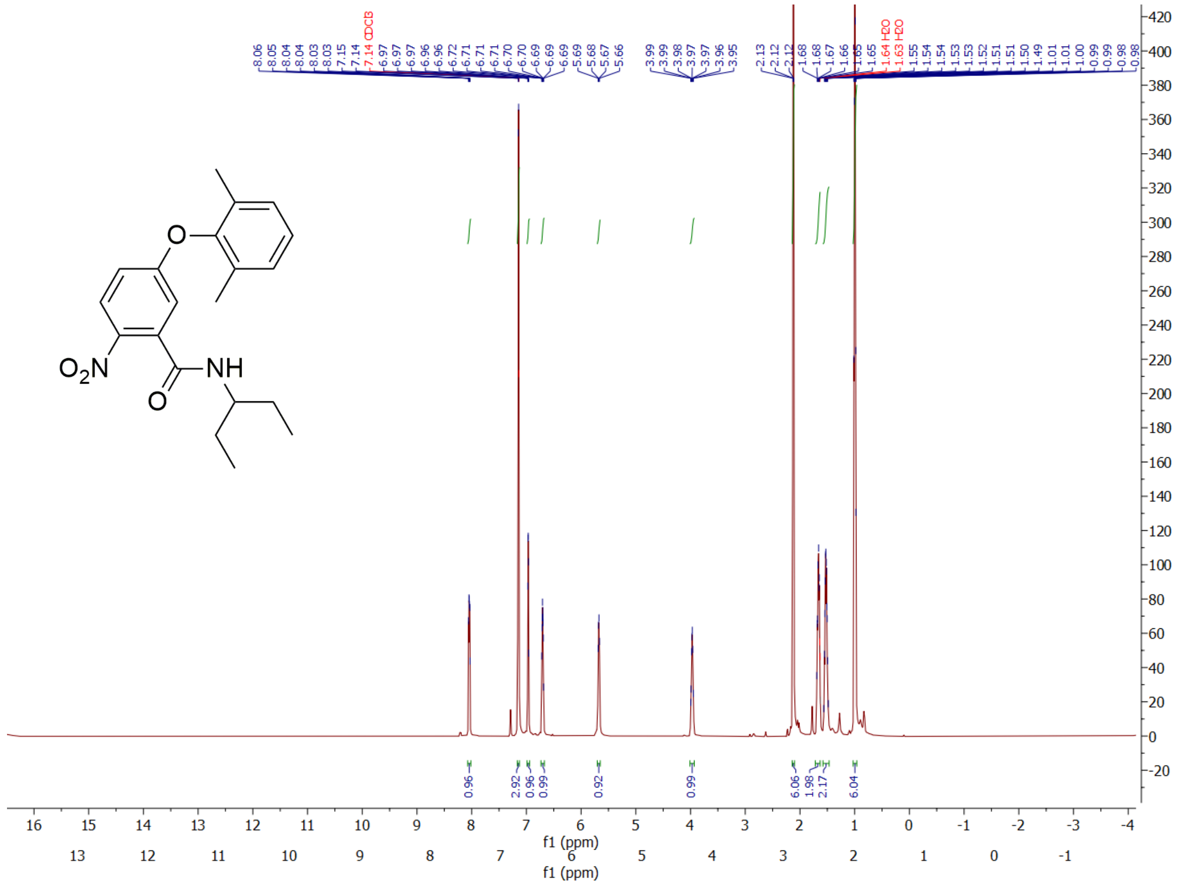

a

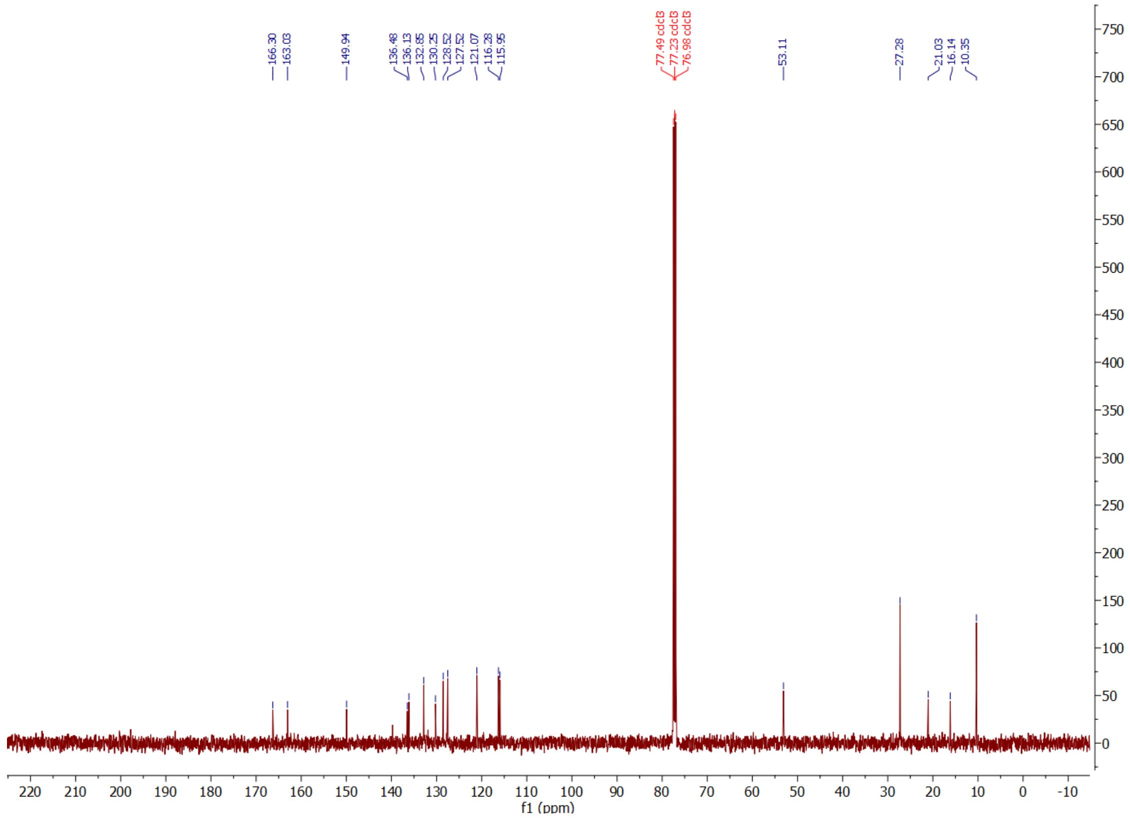

b

Figure S10. (a) ^{1}H NMR of **(10)** (600 MHz, $CDCl_3$); (b) ^{13}C NMR of **(10)** (126 MHz, $CDCl_3$).

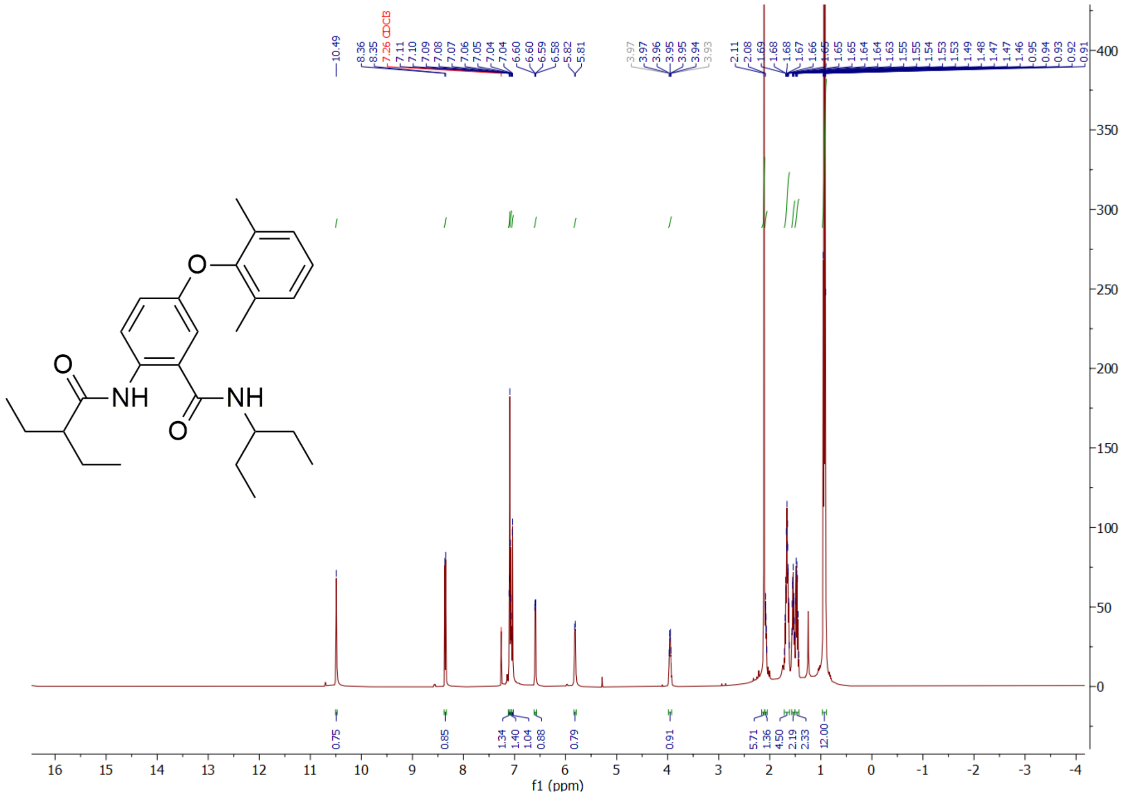

a

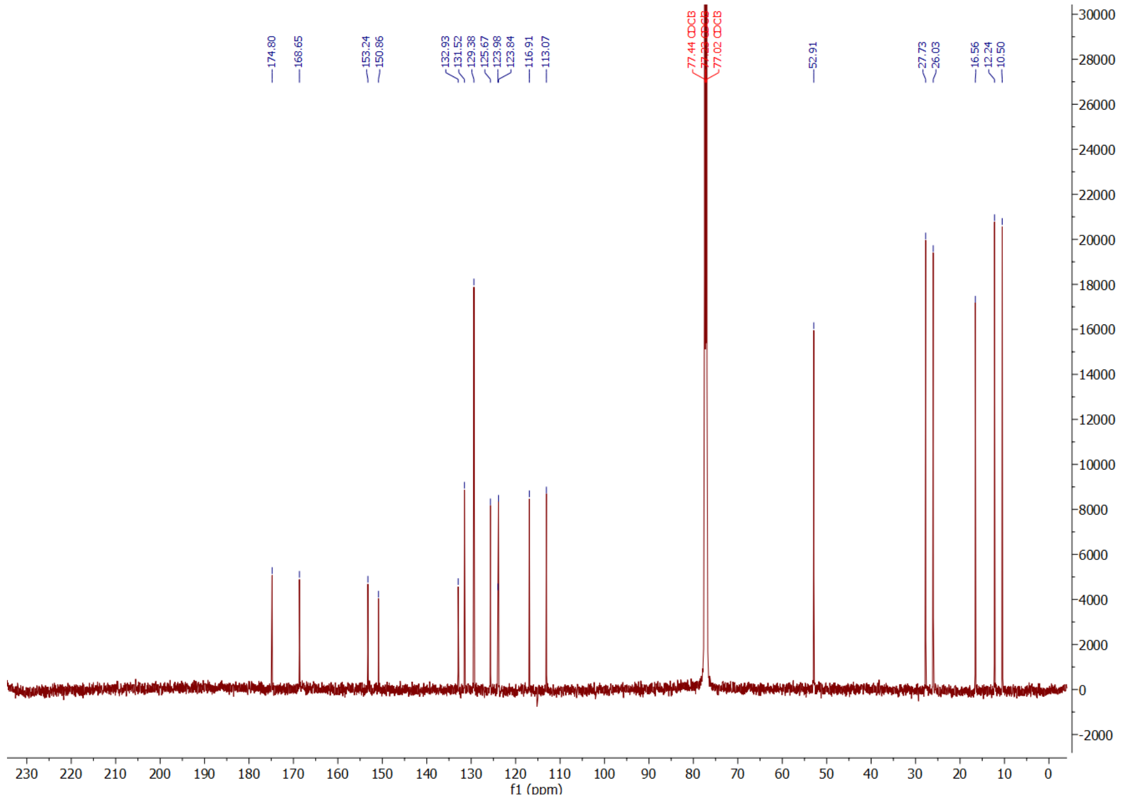

b

Figure S11. (a) ^{1}H NMR of **(Dox)** (500 MHz, $CDCl_3$); (b) ^{13}C NMR of **Dox** (151 MHz, $CDCl_3$).

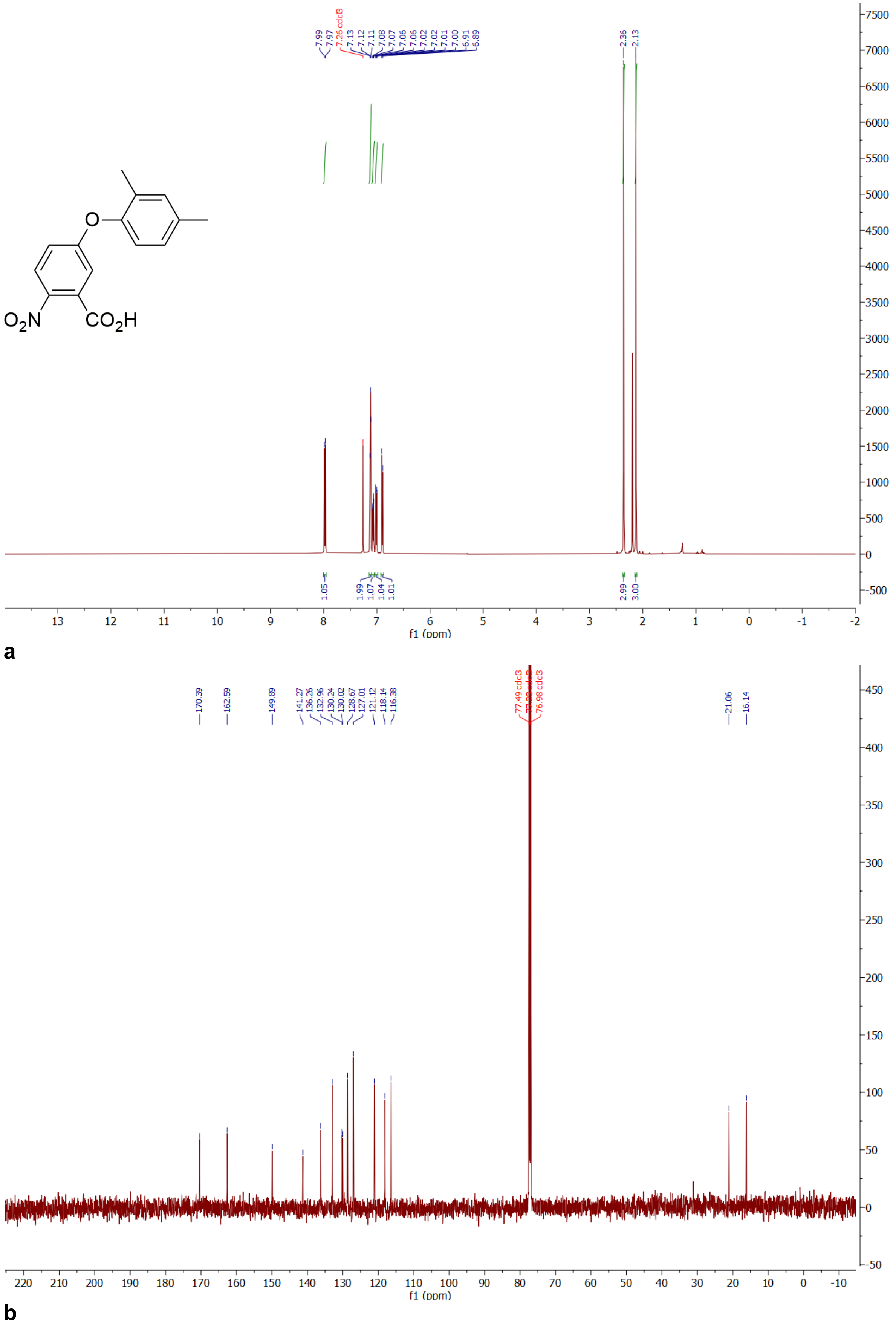

Figure S12. (a) ^{1}H NMR of **(5c)** (500 MHz, $CDCl_3$); (b) ^{13}C NMR of **(5c)** (126 MHz, $CDCl_3$).

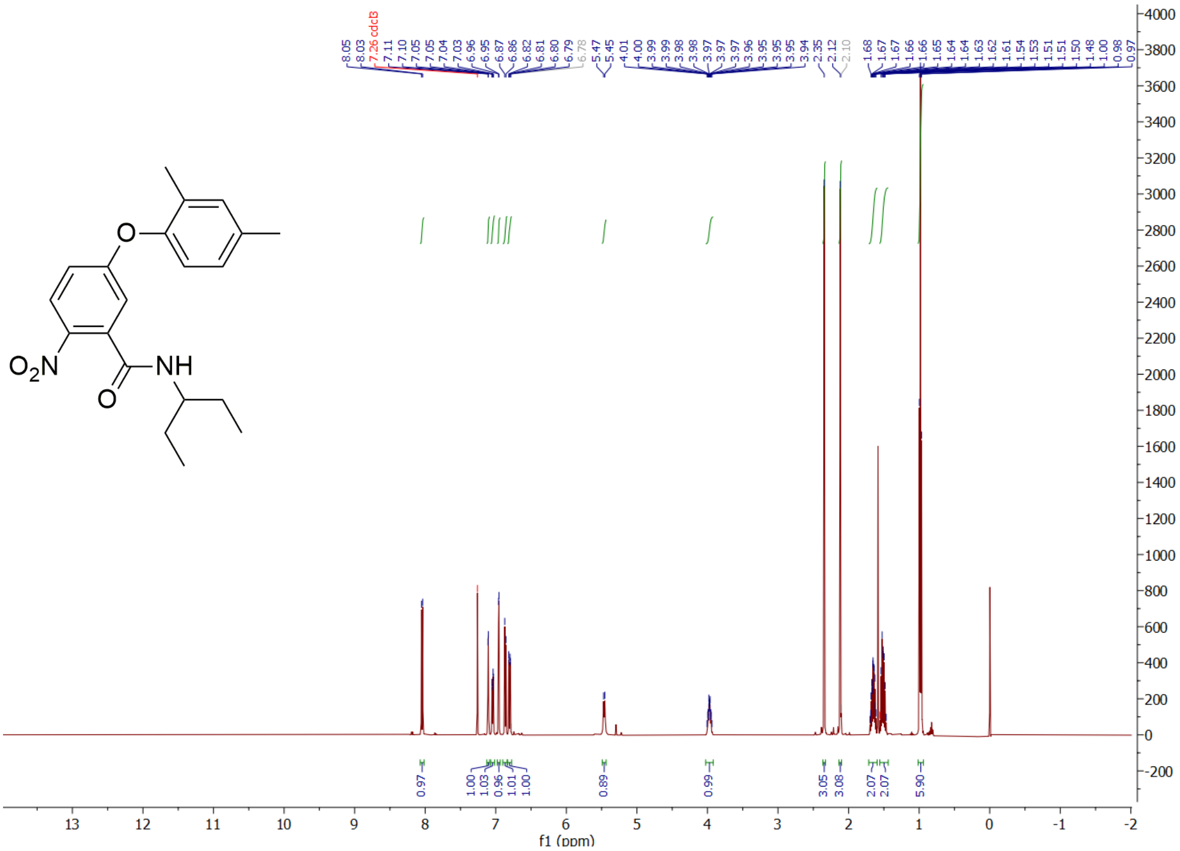

a

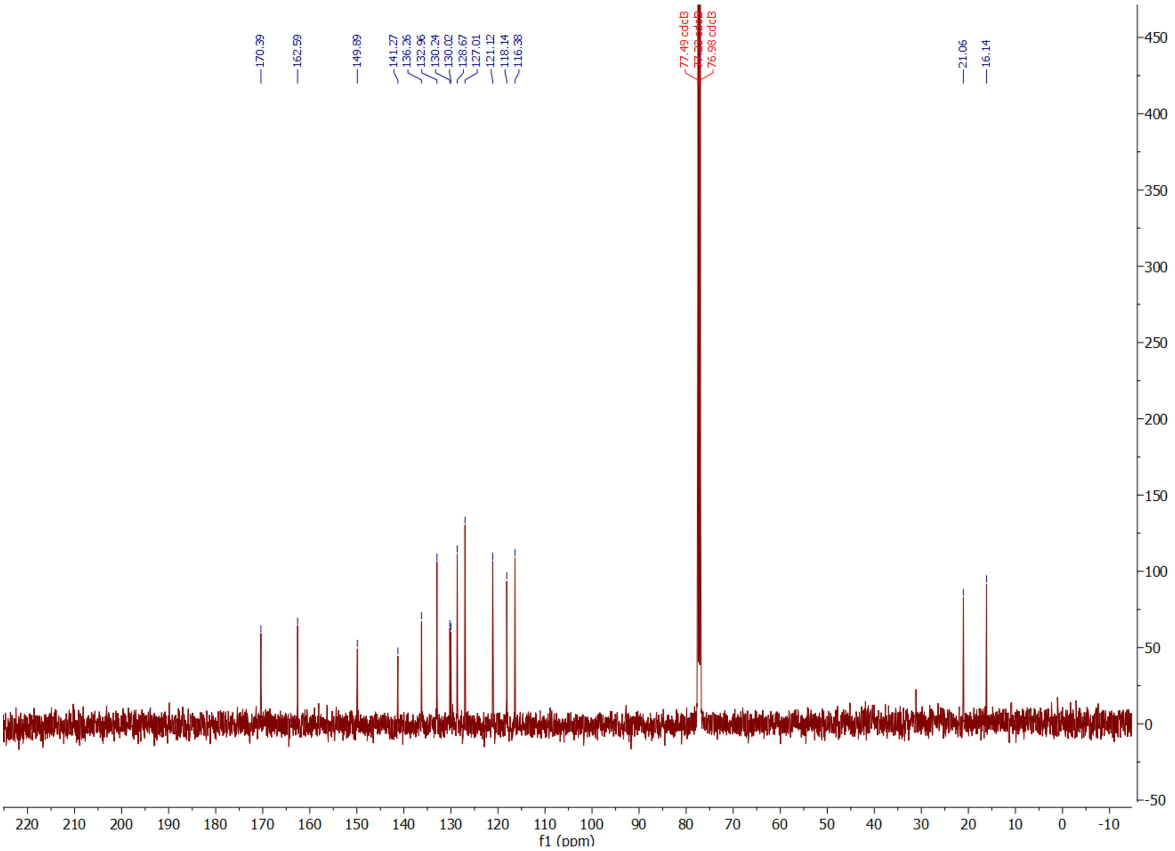

b

Figure S13. (a) ^{1}H NMR of **(12)** (500 MHz, $CDCl_3$); (b) ^{13}C NMR of **(12)** (151 MHz, $CDCl_3$).

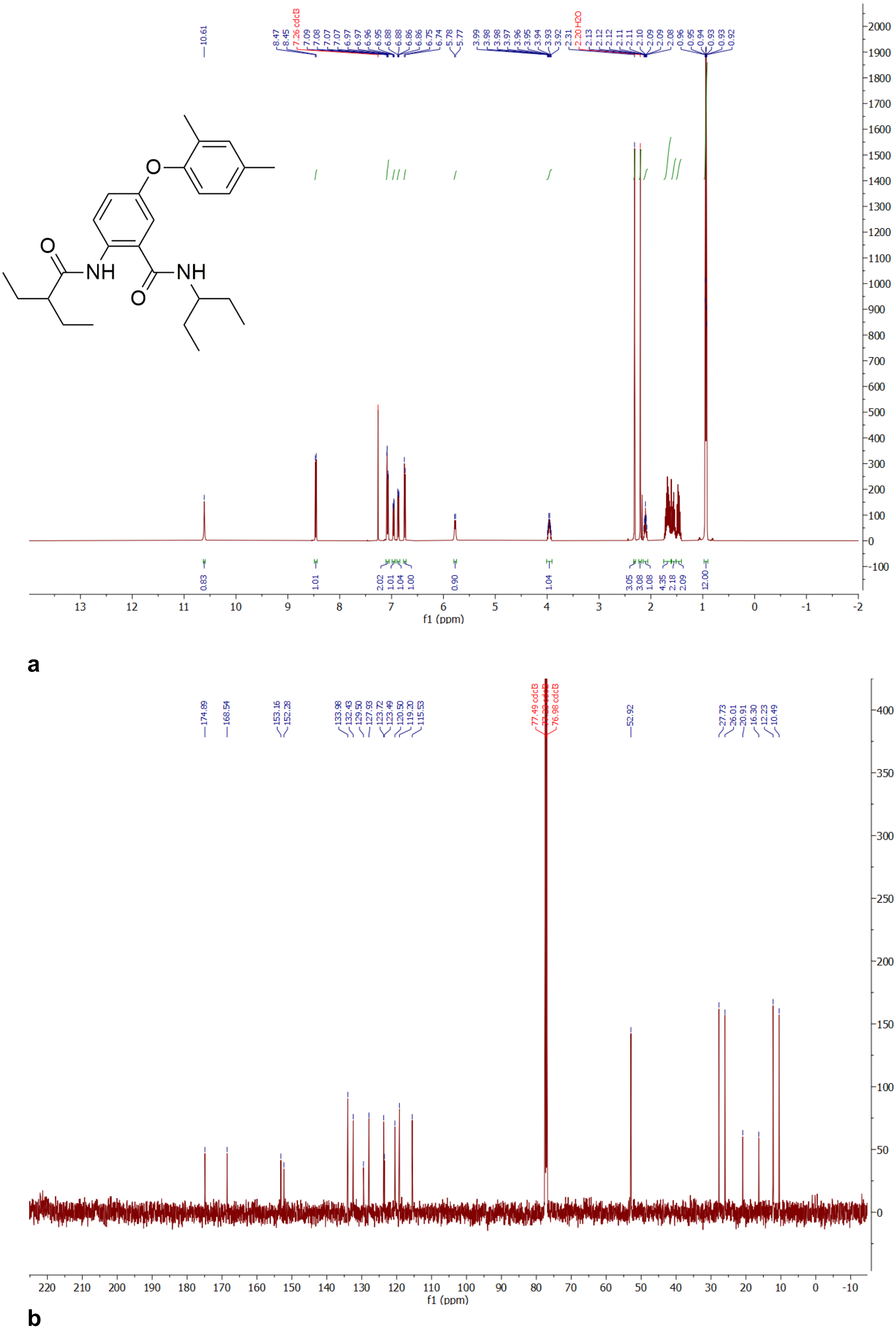

Figure S14. (a) ^{1}H NMR of **(Dox$_{24}$)** (500 MHz, $CDCl_3$); (b) ^{13}C NMR of **Dox$_{24}$** (126 MHz, $CDCl_3$).

Proofs for the selective formation of ethers, rather than esters. Etherification in 2ME selectively produces the ether derivatives of 2-nitrobenziuc acid, **3** (Scheme 1a), as one-dimensional NMR spectra and HRMS confirm. The products of etherification of the hydroxyl and esterification of the carboxyl of 5-hydroxy-2-nitrobenzoic acid, however, have similar ^{1}H and ^{13}C NMR spectra and identical exact masses obtainable by HRMS analysis. Therefore, we resort to through-space correlation structural analysis, as implemented by NOESY, to examine if products **3** are ethers or esters. For comparison, we prepare the disubstituted (ether, ester) derivatives, **2** (Scheme 1), using conventional heating.

The NOESY spectrum of the product, **3a**, from reacting **1** with *n*-butylhalides in 2ME under microwave radiation (Scheme 1a, S1) shows correlations between the α-proton of the butyl chain, b_1, and the anthranilic protons at positions 4 and 6, a_4 and a_6 (Chart S1a, Figure S15). A through-space NOE correlation between a_6 and b_1 (Chart S1a) is, indeed, plausible regardless if the butyl is coupled as ether at position 5 or as ester with the carboxyl at position 1. Observing an NOE correlation between a_4 and b_1 (Chart S1a, Figure S15), however, is highly unlikely if the butyl is bonded to the carboxyl as an ester. Therefore, the through-space correlation between a_4 and b_1 suggests that treating 2-nitrobenzoic acid, **1**, with alkylhalide in 2ME leads to selective formation of ether derivatives, **3** (Scheme 1a, S1)

To further assure that the products from reacting **1** with alkylhalides in 2ME leads to ethers, rather than esters, we undertake NOE analysis of ether ester derivative, **2**, with R = n-C_4H_9. The NOESY spectra of **2a** reveal that both α-butyl protons, b_1 and c_1, show strong correlations with the other alkyl protons, but only one of them correlates with aril protons a_4 and/or a_6 (Chart S1, Figure S16). These findings are consistent with assigning the proton that correlates with a_4 and a_6 to the α-butyl protons to the ether alkyl chain. The other α-butyl protons belong to the ester alkyl chain (Chart S1b). It suggests that most prevailing conformer of **2a** is the one in which the butyl of the ester is pointing away from the aromatic ring, and the relaxation of c_1 through c_2 and c_3 dominates, making the NOE correlation between c_1 and a_6 (Figure S16b).

Reacting **3** with amines, under carboxyl-activating conditions, produces amide with good yields, e.g., **10** (Scheme S2). Esters with free hydroxyl groups cannot produce amides under such conditions. Therefore, **3** must have free carboxylate and the butyl chain has to be attached to the hydroxyl at position 5 in order obtaining amides, such as 10, to be possible. That is, the products **3** are ether derivatives.

Chart S1. Structures of **(3a)** and **(2a)** with key through-space correlations obtained from the NOESY analysis (Figure S15b, S16b).

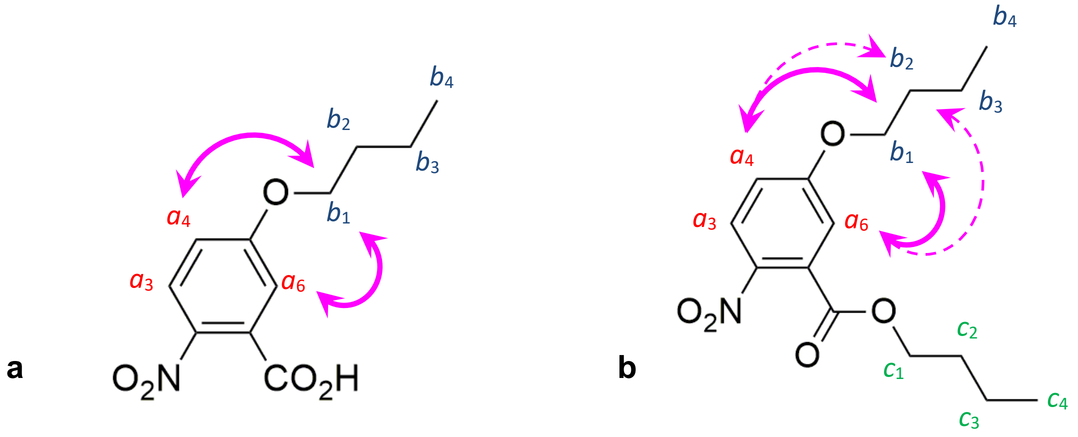

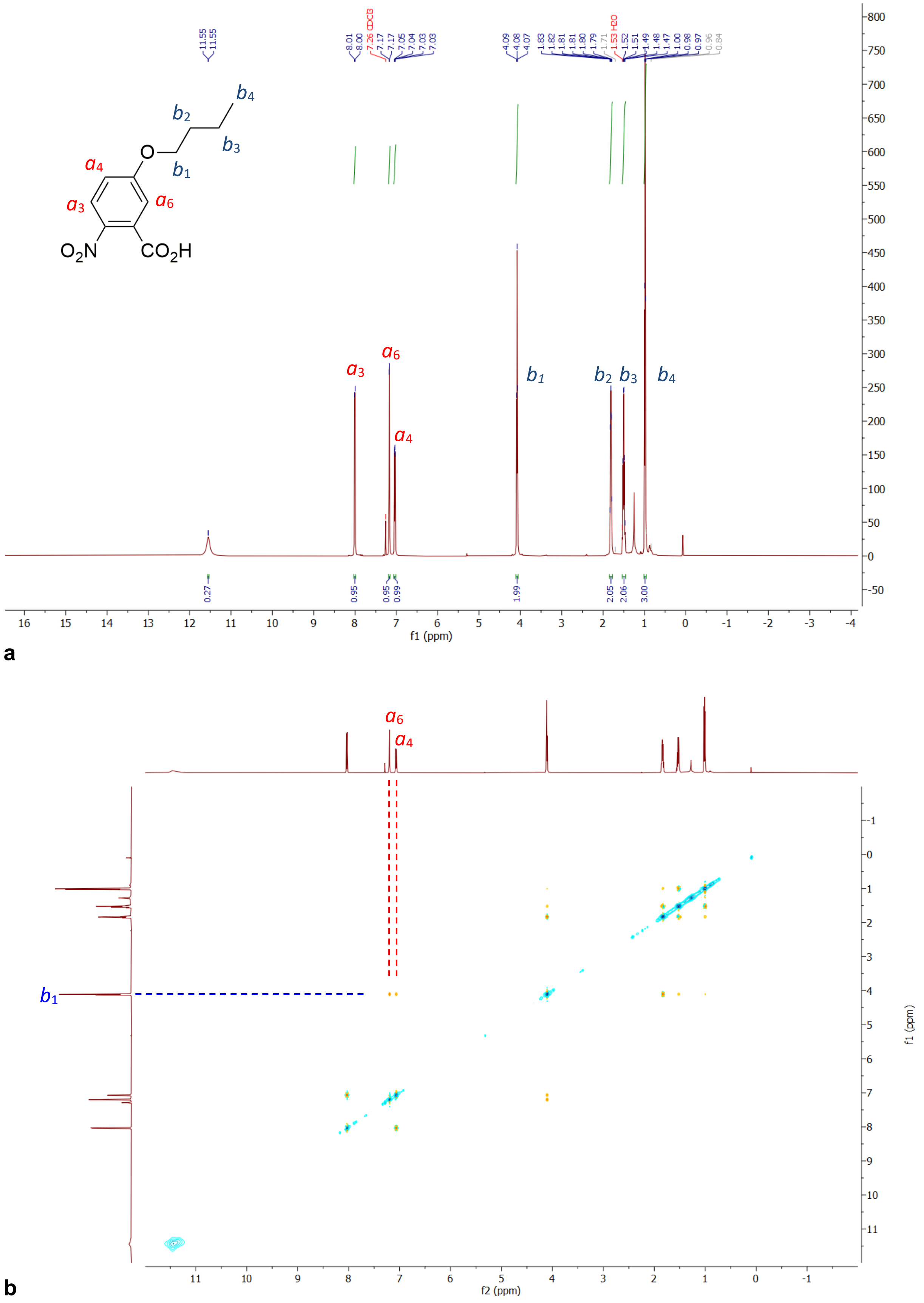

Figure S15. (a) ^{1}H NMR and (b) NOESY spectra of **(3a)** (600 MHz, $CDCl_3$).

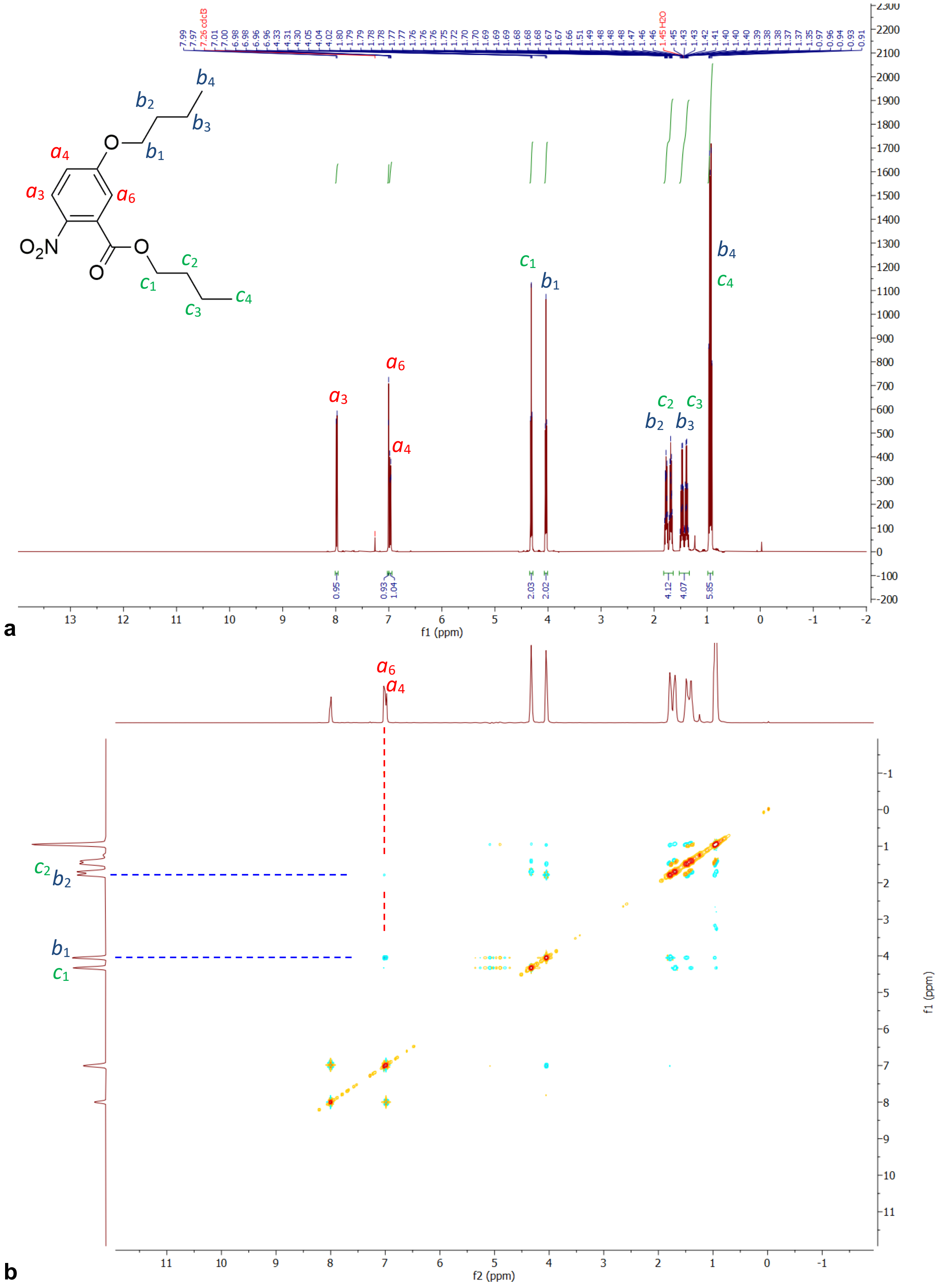

Figure S16. (a) ^{1}H NMR and (b) NOESY spectra of **(2a)** (500 MHz, $CDCl_3$).

***Syn* and *anti* conformers of Dox.** DFT computational analysis reveals that *anti* conformers of the phenoxy Aa residues are energetically slightly more stable than the *syn* conformers (Table S3). This finding is consistent with the steric hindrance between the phenoxy side chains at position 5 and the amide at position 1 (Figure S16). Nevertheless, the energy difference between the *syn* and *anti* conformers of Dox, $\Delta\mathscr{E}$(Dox), is smaller that $k_B T$ for room temperature. Furthermore, polarizable and polar solvating media depletes these energy differences, i.e., $\Delta\mathscr{E}$(Dox) → 0 (Table S3). These findings indicate that at room temperature both conformers of Dox should be present in significant amounts under thermodynamic equilibrium. For the gas phase, for example, $\Delta\mathscr{E}$(Dox) = 17 meV that indicates for 66% *anti* and 34% *sin* conformers of Dox. The solvent-induced decrease in $\Delta\mathscr{E}$(Dox) decreases the difference between the equilibrium concentrations of the two conformers to less than 20% difference, i.e., |[*anti*] / [*syn*]| < 1.5. Oxidizing the phenoxy Aa residues slightly increases the difference between the energy of *syn* and *anti* conformers, as apparent from the differences between the ionization energies, ΔIE, that are larger than $\Delta\mathscr{E}$(Table S3).

Despite the computationally predicted abundance of both *anti* and *syn* conformers (Table S3), the ^{1}H and ^{13}C NMR spectra of Dox reveal a single set of chemical shifts for the methyls at positions 2 and 6, for the protons and the carbons at positions 3 and 5, and for the carbons at position 2 and 6 of the 2,6-dimethylphenyloxy group (Figure S11). These results suggest that (1) either the synthesis produced only one of the *syn* or *anti* isomer of Dox that remains conformationally locked; or (2) the exchange between the *syn* and *anti* conformers is faster than the NMR acquisition timescales. NOESY spectra of Dox do not reveal any though-space correlations between the phenyloxy protons at positions 3, 4 and 5 and the other protons of the Dox molecule. This NOESY result, therefore, does not provide information about the conformer(s) present in the Dox solutions. Mechanistic consideration of the nucleophilic aromatic substation used for preparing the precursor for Dox suggest that the only plausible way for obtaining only a single isomers, *anti*, is if (1) phenolate attack the aromatic carbon (to replace the fluorine) equatorially, with the two six-member rings orthogonal to each other; and (2) the carboxylate completely prevents the attack from the direction of position 6 that produces the *syn* conformer. Both *syn* and *anti* conformers, however, are present in considerable amounts (Table S3), suggesting that the amide or carboxyl group at position 1 cannot completely prevent the Dox precursors from assuming *syn* geometry. That is, the carboxyl group, however, is not large enough to completely prevent such attack and the *syn* conformer should still form in detectable amounts. Because the NMR spectra do not reveal two separate isomers, we conclude that the *syn* and *anti* Dox interexchange and these conformational transformation occur in the millisecond range or faster.

Table S3. Differences between the energies of the *syn* and *anti* conformers of the Aa ether derivatives.

	$\Delta\mathscr{E}$(Aa) / eV [a]			ΔIE / eV [b]		
	gas phase	CH_2Cl_2 [c]	CH_3CN [c]	gas phase	CH_2Cl_2 [c]	CH_3CN [c]
**Fox**	< 0.010	< 0.010	< 0.010	0.050	0.015	0.010
**Dox**	0.017	< 0.010	< 0.010	0.042	0.013	< 0.010
**Dox_{24}**	0.014	< 0.010	< 0.010	0.064	0.027	0.020

[a] $\Delta\mathscr{E} = \mathscr{E}(syn) - \mathscr{E}(anti)$: The $\mathscr{E}(syn)$ and $\mathscr{E}(anti)$ are of the DFT-optimized structures of the ground-states of the conformers. Hartree-Fock calculations also yield $\mathscr{E}(syn) > \mathscr{E}(anti)$, but with $\Delta\mathscr{E} \lesssim 5$ meV for all cases; [c] From the computed ionization energies (IE) of the conformers, i.e., ΔIE = IE(*syn*) – IE(*anti*). Using Koopmans' theorem allows for estimating IE from the difference between the computed energies of the doublet (singly oxidized) and the ground-state singlet states. [d.] Implemented using polarizable continuum model (PCM).

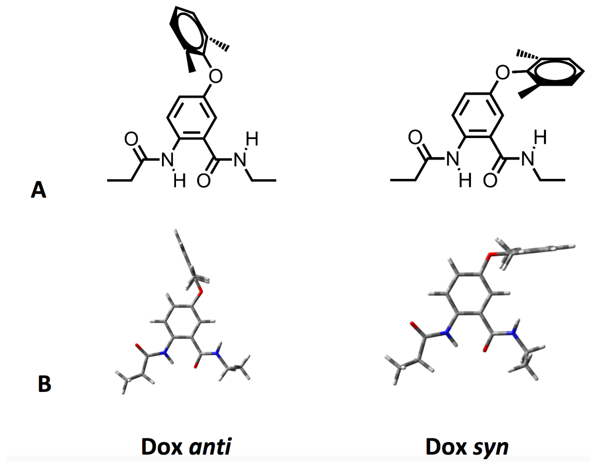

Figure S17. Representation of the *anti* and *syn* conformers of Dox using (A) ChemDraw and (B) Gaussview of the DFT optimized structures using the tube view.

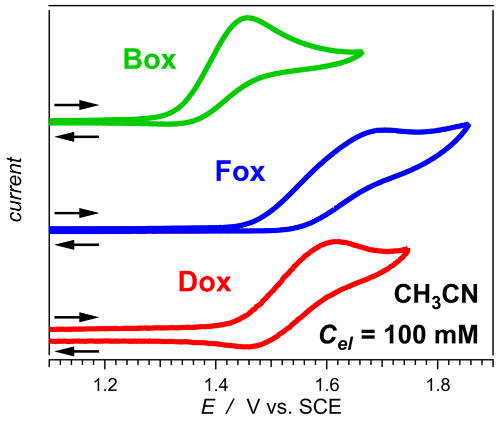

Figure S18. Cyclic voltammograms of Aa ether residues (Chart 1b-e) for acetonitrile in the presence of 100 mM electrolyte, $N(n\text{-}C_4H_9)_4PF_6$, at scan rate, v = 0.1 V s^{-1}, showing irreversible oxidation of Box and Fox and partial reversibility of the oxidation of Dox.

METHODS

Cyclic voltammetry. Electrochemical studies are conducted using Reference 600™ Potentiostat/Galvanostat/ZRA (Gamry Instruments, PA, U.S.A.), connected to a three-electrode cell. Glassy carbon electrode and platinum wire serve as working and counter electrode, respectively. The reference saturated calomel electrode (SCE) is connected with the cell via a salt bridge filled with 100 mM $N(n\text{-}C_4H_9)_4PF_6$ solution in acetonitrile. Using such electrolyte solution in the bridge that is missile with aqueous and non-polar organic solvents aids with challenging potential drops at the junctions. To correct for potential drifts in the reference electrode (which is SCE, connected with the cell via a salt bridge), ferrocene is used as a standard ($E^{(1/2)}$ = 0.45 ± 0.01 V vs. SCE for CH_3CN, 100 mM $N(n\text{-}C_4H_9)_4BF_4$). Voltammograms of the standard are recorded before and after each set of measurements. Anhydrous aprotic solvents with different polarity are employed with different concentrations of supporting electrolyte, $N(n\text{-}C_4H_9)_4PF_6$. Specifically, we employ dichloromethane (Figure 1) and acetonitrile (Figure S18) for this study. Prior to recording each voltammogram, each sample is extensively purged with high-purity argon while maintaining its volume constant by adding more of the anhydrous solvent. For each solvent, a set of voltammograms is recorded where the electrolyte concentration is increased from 25 mM to 200 mM in increments of 25 mM. The half-wave potentials, $E^{(1/2)}$, are determined (1) from the

midpoints between the cathodic and anodic peak potentials, E_c and E_a, respectively, for reversible or quasireversible voltammograms; and (2) from the inflection points of the waves for irreversible oxidation and reduction. The values of E_a and E_c are determined from the zero points of the first derivatives of the voltammograms, i.e., the potentials where $\partial I / \partial E = 0$ at $\partial E / \partial t$ = constant. The inflection points are determined from the zero point of the second derivatives of the voltammograms, $\partial^2 I / \partial E^2 = 0$ at $\partial E / \partial t$ = constant. The second derivatives of reversible and quasi-reversible voltammograms show that the inflection-point potentials are quite close to the mid-points between E_a and E_c, ensuring the reliability for the estimates of $E^{(1/2)}$ from the inflection points of irreversible voltammograms. The voltammograms are recorded at a scan rate from 50 to 750 mV s^{-1}. For each solvent, the dependence of $E^{(1/2)}$ on the electrolyte concentration, C_{el}, allows for extrapolating the values of the potentials for $C_{el} = 0$, i.e., the values of the potentials for the neat solvent.

Steady-state optical spectroscopy. UV/visible absorption spectra were recorded in a transmission mode using a JASCO V-670 spectrophotometer (Tokyo, Japan); and steady-state emission spectra were measured, also in a transmission mode, with a FluoroLog-3 spectrofluorometer (Horiba-Jobin-Yvon, Edison, NJ, USA). Despite the noticeably large Stokes' shifts (Table S4), the wavelength of the crossing point of the normalized absorption and emission spectra, λ_{00}, provide estimates for the optical excitation energy, $\mathscr{E}_{00} = h\, c / \lambda_{00}$. (Figure S19, Table 2).

Table S4. Absorption and emission maxima of the ether Aa derivatives (Chart 1b-e).

	CH_2Cl_2		CH_3CN	
	$\lambda_{abs}^{(max)}$ / nm	$\lambda_{fl}^{(max)}$ / nm	$\lambda_{abs}^{(max)}$ / nm	$\lambda_{fl}^{(max)}$ / nm
**Box**	320	399	318	397
**Fox**	314	396	311	394
**Dox**	315	396	315	395
**Dox_{24}**	317	397	314	395

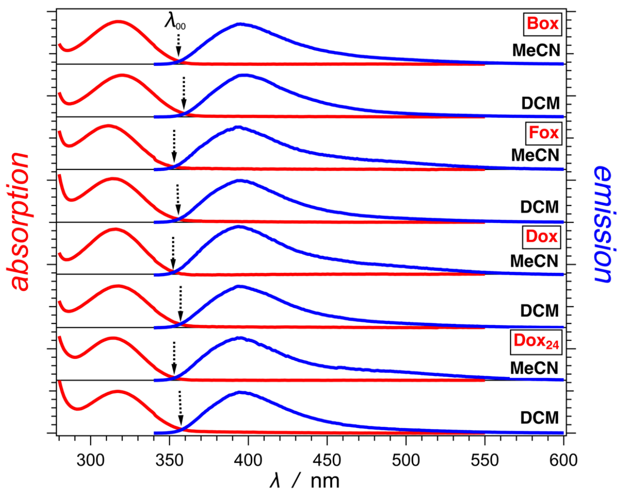

Figure S19. Optical spectra of the ether Aa derivatives (Chart 1b-e) for acetonitrile (MeCN) and dichloromethane (DCM). The dotted arrows represent the wavelengths, λ_{00}, corresponding the zero-to-zero transition energies, $\mathscr{E}_{00}$ (Table 1).

Computational methods. The *N*-acylated Aa ether residues are modeled using density functional theory (DFT). The alkyl chains at the C- and N- termini are truncated to ethyls. The DFT calculations are performed at the B3LYP/6-311+G(d,p) level of theory, for the gas phase, using Gaussian 09, leading to convergence to the *syn* and *anti* conformers (Figure S17). Spin-unrestricted calculations are used for radical-cation (doublet state) modeling (Figure 2, S20). We report frontier-orbital distributions for the Aa ether residues in the gas phase (Figure S21). Solvent effects were estimated by comparing the results from gas-phase calculations to those in an integral equation formalism polarizable continuum model (PCM). Basis on the Koopmans' theorem, we use the difference between the energies of the radical cation doublet states and the ground states of each of the conformers for estimating the ionization energy (IE) of the *syn* and *anti* conformers of the Aa residues.

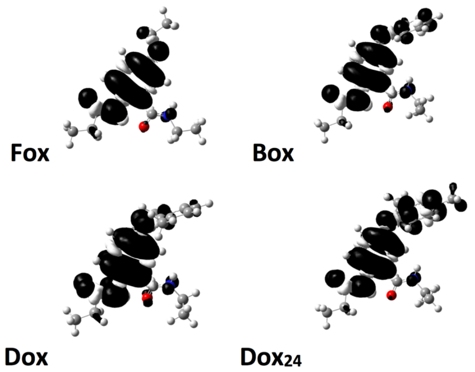

Figure S20. Spin density distributions (SDDs) of the radical cation showing the delocalization of the positive charge of the Aa ether residues (*syn* conformers) using DFT calculations. The alkyl chains from the C- and N- termini are truncated to ethyls for the computational studies.

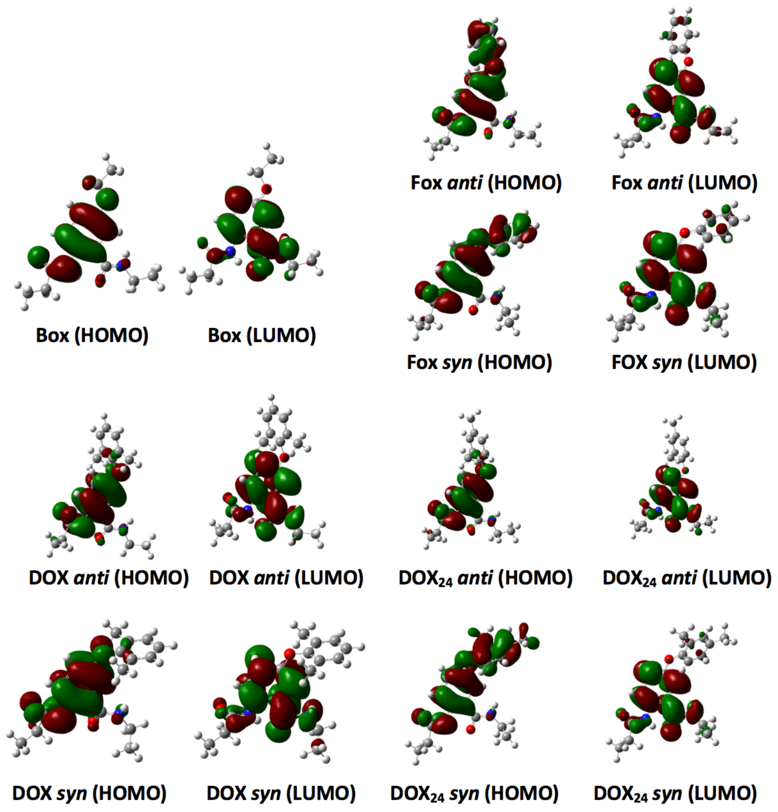

Figure S21. Frontier orbitals of the Aa ether residues (*syn* and *anti* conformers) for the gas phase, obtained using DFT calculations. The alkyl chains at the C- and N-termini are truncated to ethyls for the computational studies.
